# Robustness of STDP to spike timing jitter

**DOI:** 10.1038/s41598-018-26436-y

**Published:** 2018-05-25

**Authors:** Yihui Cui, Ilya Prokin, Alexandre Mendes, Hugues Berry, Laurent Venance

**Affiliations:** 1 Dynamics and Pathophysiology of Neuronal Networks Team, Center for Interdisciplinary Research in Biology (CIRB), College de France, CNRS, INSERM, PSL Research University, Paris, France; 2 INRIA, Villeurbanne, France; 3 University of Lyon, LIRIS UMR5205 Villeurbanne, France

## Abstract

In Hebbian plasticity, neural circuits adjust their synaptic weights depending on patterned firing. Spike-timing-dependent plasticity (STDP), a synaptic Hebbian learning rule, relies on the order and timing of the paired activities in pre- and postsynaptic neurons. Classically, in *ex vivo* experiments, STDP is assessed with deterministic (constant) spike timings and time intervals between successive pairings, thus exhibiting a regularity that differs from biological variability. Hence, STDP emergence from noisy inputs as occurring in *in vivo*-like firing remains unresolved. Here, we used noisy STDP pairings where the spike timing and/or interval between pairings were jittered. We explored with electrophysiology and mathematical modeling, the impact of jitter on three forms of STDP at corticostriatal synapses: NMDAR-LTP, endocannabinoid-LTD and endocannabinoid-LTP. We found that NMDAR-LTP was highly fragile to jitter, whereas endocannabinoid-plasticity appeared more resistant. When the frequency or number of pairings was increased, NMDAR-LTP became more robust and could be expressed despite strong jittering. Our results identify endocannabinoid-plasticity as a robust form of STDP, whereas the sensitivity to jitter of NMDAR-LTP varies with activity frequency. This provides new insights into the mechanisms at play during the different phases of learning and memory and the emergence of Hebbian plasticity in *in vivo*-like activity.

## Introduction

Long-term changes in synaptic efficacy between neurons are thought to underlie learning and memory^1^ and can be assessed with a synaptic Hebbian paradigm such as spike timing-dependent plasticity (STDP)^2–8^. In STDP, the occurrence of timing-dependent-long-term potentiation (tLTP) or -depression (tLTD) is the result of three factors. The two first ones are the two coincident activity patterns on either side of the synapse, which depends on (1) the relative timing between pre- and postsynaptic spikes (Δ*t*_STDP_)^4,8^, (2) the dendritic and axonal propagation delays^9^, (3) the number of paired spikes (*N*_pairings_)^10–13^, (4) the frequency of the paired spikes (*F*_pairings_)^6,7,10,11,14^ and (5) membrane depolarization^14,15^; the third factor involves in STDP expression mainly refers to neuromodulators^16,17^ and glial cells^18^.

STDP has been attracting a lot of interest in computational and experimental neurosciences because it relies on spike correlation and has emerged as a candidate mechanism for experience-dependent changes in neural circuits, including map plasticity^8,19–23^. STDP is classically investigated using regular repetitions of the same spike timing and fixed intervals between successive paired stimulations. A typical experimental protocol consists in pairing pre- and postsynaptic stimulations with a fixed Δ*t*_STDP_ (ranging from −30 to +30 ms for plasticity induction); Δ*t*_STDP_ < 0 when the postsynaptic stimulation occurs before the paired presynaptic one (post-pre pairings), whereas Δ*t*_STDP_ > 0 when the presynaptic stimulation occurs before the postsynaptic one (pre-post pairings). These pairings are then repeated between 15 and 200 times (between 0.1 and 5 Hz) with the spike timing and the time interval between successive pairings being kept constant.

Regular stimulation paradigms produce patterns of activity that are likely to differ from the variability expected in *in vivo*-like firing. *In vivo* paired natural visual stimulations with postsynaptic action potential lead to Hebbian STDP with specific characteristics when compared to *ex vivo* STDP (broader membrane potential changes and STDP window), which can be explained by jittered presynaptic inputs occurring *in vivo*^22^. A theoretical study has started to explore naturalistic stimulations and showed that when neurons fire irregularly, the impact of spike timing in plasticity becomes weaker than the influence of the firing rate^24^. However, whether STDP emergence and maintenance is robust against biological variability remains to be investigated. To address this question, it is important to take into account various forms of STDP, involving distinct intracellular signal transduction pathways, i.e. NMDAR-, mGluR- or endocannabinoid(eCB)-mediated STDP^25–29^ (for reviews see^8,30,31^). It is thus expected that those various STDP forms might exhibit different robustness to spike train variability.

Here, we show with patch-clamp recordings and mathematical modeling that corticostriatal NMDAR- and eCB-STDP do not exhibit the same sensitivity to noisy spike timings: eCB-plasticity (eCB-tLTD and eCB-tLTP) appeared robust to jittering whereas NMDAR-tLTP was fragile. However, increasing the number or frequency of pairings improved NMDAR-tLTP robustness. Our results further indicate that the robustness of NMDAR-tLTP to jitter is also strengthened by the irregularity of the spike-train stimulations.

## Results

Corticostriatal synapses exhibit a bidirectional STDP in which NMDAR-tLTP, eCB-tLTD^29,32–34^ or eCB-tLTP^12,13^ are induced depending on the number of pairings (*N*_pairings_) and the spike timing (Δ*t*_STDP_). While STDP is classically investigated using fixed Δ*t*_STDP_, we examine here the effect of noisy Δ*t*_STDP_, closer to *in vivo*-like firing. Here, we define the Inter-Pairing-Interval (IPI) as the time interval between two consecutive presynaptic stimulations, whereas Δ*t*_STDP_ is the time interval between the post- and the presynaptic stimulation times within a given paired stimulation (in a “standard” STDP protocol both IPIs and Δ*t*_STDP_ are constant).

### NMDAR- and eCB-mediated STDP triggered with fixed Δt_STDP_

**Figure 1:**
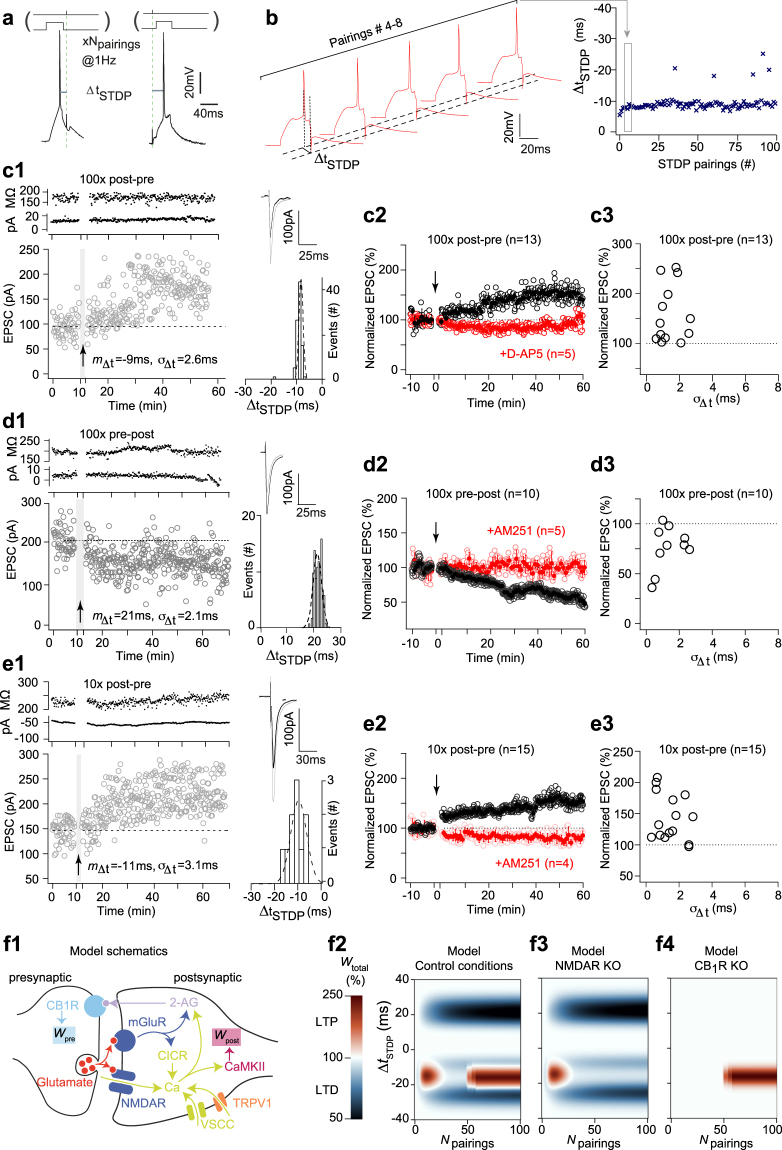
NMDAR- and eCB-mediated STDP induced with fixed Δ*t*_STDP_. (**a**) STDP protocol: a single postsynaptic spike was paired with a single cortical stimulation, 10 or 100 times at 1 Hz. (**b**) Example of 5 successive pairings (#4–8, from c1) exhibiting relatively fixed Δ*t*_STDP_ (#4–8: −8.0, −7.8, −7.4, −8.8 and −8.0 ms). Plotting the successive Δ*t*_STDP_ illustrates their low variance *σ*_Δ*t*_ (2.6 ms). (**c**) Corticostriatal NMDAR-tLTP induced by 100 post-pre pairings with fixed Δ*t*_STDP_. (**c1**) Example of tLTP induced by 100 post-pre pairings (*m*_Δ*t*_ = −9 ms, *σ*_Δ*t*_ = 2.6 ms). The mean baseline EPSC amplitude, 95 ± 3 pA, increased by 77% to 168 ± 6 pA 45 minutes after pairings. Upper panel, time course of Ri (baseline: 172 ± 2 MΩ and 35–45 min after pairings: 170 ± 3 MΩ; change of 1%) and holding current (I_hold_). Inset: distribution of the 100 Δ*t*_STDP_. (**c2**) Averaged time-courses of tLTP induced by 100 post-pre pairings; this tLTP was NMDAR-mediated because prevented by D-AP5 (50 μM). (**c3**) Relationship between the STDP magnitude and the STDP jitter for each of the recorded neurons. (**d**) Corticostriatal eCB-tLTD induced by 100 pre-post pairings with fixed Δ*t*_STDP_. (**d1**) Example of tLTD induced by 100 pre-post pairings (*m*_Δ*t*_ = +21 ms, *σ*_Δ*t*_ = 2.1 ms); the mean baseline EPSC amplitude, 206 ± 4 pA, decreased by 25%, to 154 ± 8 pA, 45 minutes after pairings. Upper panel, time course of Ri (baseline: 195 ± 1 MΩ and 45–55 min after pairings: 196 ± 2 MΩ; change <1%) and I_hold_. Inset: distribution of the 100 Δ*t*_STDP_. (**d2**) Averaged time-courses of tLTD induced by 100 pre-post pairings; this tLTD was CB_1_R-mediated because prevented by AM251 (3 μM). (**d3**) Relationship between the STDP magnitude and the jitter. (**e**) Corticostriatal eCB-tLTP induced by 10 post-pre pairings with fixed Δ*t*_STDP_. (**e1**) Example of tLTP induced by 10 post-pre pairings (*m*_Δ*t*_ = −11 ms, *σ*_Δ*t*_ = 3.1 ms); the mean baseline EPSC amplitude, 147 ± 3 pA, increased by 43% to 209 ± 9 pA 45 minutes after pairings. Upper panel, time course of Ri (baseline: 227 ± 2 MΩ and 45–55 min after pairings: 239 ± 2 MΩ; change of 5%) and I_hold_. Inset: distribution of the 10 Δ*t*_STDP_. (**e2**) Averaged time-courses of tLTP induced by 10 post-pre pairings; this tLTP was CB_1_R-mediated because prevented by AM251. (**e3**) Relationship between the STDP magnitude and the jitter. (**c1**-**d1**-**e1**) Insets: average of 12 successive EPSC amplitude at baseline (black traces) and at 40–50 min after pairings (grey traces). (**f**) Predictions of the mathematical model in the absence of added jitter. The model expresses the biochemical pathways schematized in (**f1**). (**f2**) Changes of *W*_total_ with various Δ*t*_STDP_ and *N*_pairings_. (**f3**) Removing NMDAR/CaMKII signaling suppresses the tLTP observed for Δ*t*_STDP_ > 0 and *N*_pairings_ > 50. (**f4**) Removing CB_1_R suppresses the tLTP observed for Δ*t*_STDP_ > 0 and *N*_pairings_ < 20 and the tLTD observed for Δ*t*_STDP_ < 0.

We observed bidirectional asymmetric STDP in MSNs (Fig. 1a): 100 post-pre pairings (Δ*t*_STDP_ < 0) induced tLTP whereas 100 pre-post pairings (Δ*t*_STDP_ > 0) induced tLTD. An example of the tLTP induced by 100 post-pre pairings with fixed Δ*t*_STDP_ (Fig. 1b) is illustrated in Fig. 1c1. The input resistance Ri remained stable over this period. Although we fixed the values of Δ*t*_STDP_, they slightly vary from one pairing to another (Fig. 1b). This low jitter can be written formally as Δ*t*_STDP_ = *m*_Δ*t*_ + *ξ*_Δ*t*_, were *m*_Δ*t*_ is the mean spike timing and *ξ*_Δ*t*_is a random variable with mean 0 and standard deviation *σ*_Δ*t*_. Overall, 100 post-pre pairings induced tLTP (mean EPSC amplitude recorded 60 min after protocol induction: 149 ± 13% of baseline, *p* = 0.0028, *n* = 13) (Fig. 1c2). *σ*_Δ*t*_ was 1.4 ± 0.2 ms (Fig. 1c3). This tLTP was NMDAR-mediated since prevented by D-AP5 (50 μM), a NMDAR antagonist, (88 ± 12%, *p* = 0.375, n = 5; *p* < 0.0001 when compared with tLTP in control) (Fig. 1c2). Conversely, pre-post pairings induced tLTD, as shown in the example in Fig. 1d1. Overall, 100 pre-post pairings induced tLTD (76 ± 7%, *p* = 0.0070, *n* = 10) (Fig. 1d2). *σ*_Δ*t*_ was 1.4 ± 0.2 ms (Fig. 1d3). This tLTD was CB_1_R-mediated since prevented by AM251 (3 μM), a CB_1_R specific inhibitor (99 ± 2%, *p* = 0.816, n = 5; *p* < 0.0001 when compared with tLTD in control) (Fig. 1d2). We thus find an anti-Hebbian polarity for corticostriatal STDP. We previously showed that GABA operates as a Hebbian/anti-Hebbian switch at corticostriatal synapses^34,35^ and corticostriatal STDP polarity depends on the presence^32,33^ or the absence^12,29,36,37^ of GABA_A_ receptor antagonists.

Besides this bidirectional STDP (NMDAR-tLTP and eCB-tLTD) induced for 100 pairings, we reported that low numbers of pairings (*N*_pairings_ = 5–15) induce an eCB-tLTP, dependent on the activation of CB_1_R^12,13^. Figure 1e1 shows an example of tLTP induced by 10 post-pre pairings. Overall, 10 post-pre STDP pairings induced tLTP (145 ± 9%, p = 0.0005, n = 15), which was prevented by AM251 (3 μM) (76 ± 10%, p = 0.080, n = 4; *p* < 0.0001 when compared with tLTP in control) (Fig. 1e2) as recently reported^12^. *σ*_Δ*t*_ for 10 post-pre parings STDP was 1.8 ± 0.3 ms (n = 15) (Fig. 1e3). Note that there was no difference between the *σ*_Δ*t*_ measured for NMDAR-tLTP, eCB-tLTD and eCB-tLTP (one way ANOVA, p = 0.4094).

### A computer model emulates NMDAR- and eCB-mediated STDP

To help interpret our experimental results, we used a mathematical model of the signaling pathways implicated in corticostriatal STDP, including NMDAR- and CB_1_R-plasticity^12,29,32,33^ (Fig. 1f1). The model is detailed in S1 Text, with parameter values listed in S1 and S2 Tables. We include in the Supporting Information a thorough description of the mechanisms by which eCB- and NDMAR-plasticity are expressed in the model when STDP protocol (*N*_pairings_ = 100) is applied (S3 Text). Figure 1f2 shows the value of the total synaptic weight (*W*_total_) predicted by the model with various *N*_pairings_ and Δ*t*_STDP_. In agreement with the experimental data, with small *σ*_Δ*t*_ (3 ms), the model features three main plasticity regions: tLTD was observed for short pre-post pairings (0 < Δ*t*_STDP_ < +30 ms), whereas short post-pre pairings (−30 < Δ*t*_STDP_ < 0 ms) induced a first tLTP region for low numbers of pairings (3 < *N*_pairings_ < 25) and a second tLTP region emerging for *N*_pairings_ > 50. Blocking the NMDAR-CaMKII pathway in the model (Fig. 1f3) suppresses the second tLTP region whereas blocking CB_1_R activation (Fig. 1f4) prevents the expression of tLTD and of the first tLTP region. Therefore, the model emulates both CB_1_R-tLTD and -tLTP, in agreement with the eCB-tLTD and eCB-tLTP illustrated in the experimental data of Fig. 1d and e, respectively. The model also faithfully emulates the appearance of NMDAR-tLTP for *N*_pairings_ > 50.

### Predicting the effects of jittered STDP with a mathematical model

The small jitter observed in the above experiments was the result of biological variability inherent to the experimental setup. Our next goal was to extend this study to larger jitters (3 < *σ*_Δ*t*_ < 10 ms) that we controlled experimentally. We first used constant intervals between two consecutive pairings (Inter-Pairing Interval, IPI) and used the same definition as above for jittered Δ*t*_STDP_ (*Methods*): Δ*t*_STDP_ = *m*_Δ*t*_ + *ξ*_Δ*t*_, where *ξ*_Δ*t*_ is a random variable with zero mean and *m*_Δ*t*_ is the expected value of the spike timing Δ*t*_STDP_ over the *N*_pairings_. Next, we adjusted the probability distribution function and the variance of *ξ*_Δ*t*_ to perform a parametric exploration of the impact of jitter.

**Figure 2:**
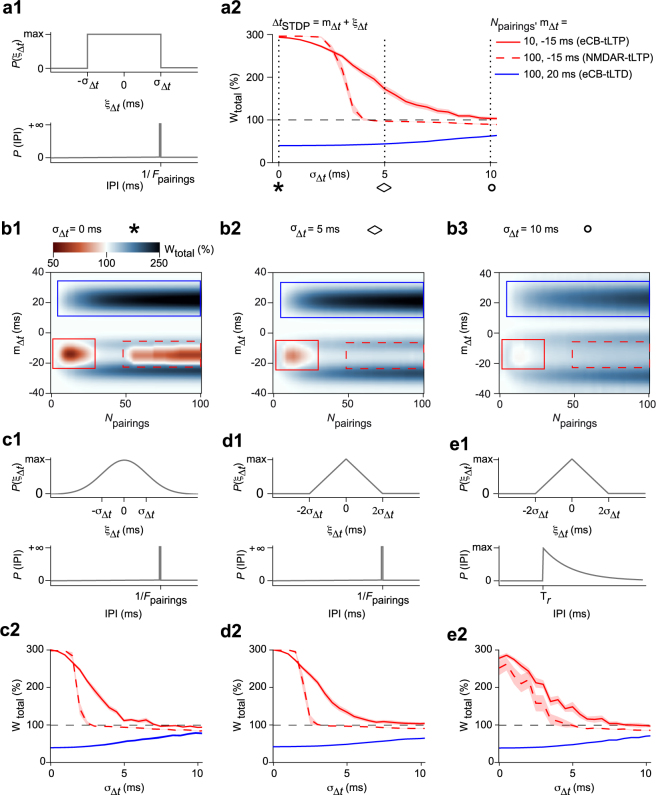
The mathematical model predicts that NMDAR-tLTP is less robust to jitter than eCB-tLTP and eCB-tLTD. (**a**) Using a jittered STDP protocol combining uniformly-distributed jitter of the spike timing with deterministic IPIs at 1 Hz (**a1**), the amplitudes of NMDAR-tLTP, eCB-tLTD and eCB-tLTP decrease with increasing jitter amplitudes *σ*_Δ*t*_ (**a2**). eCB-tLTP (red full line) was triggered with 10 pairings of average spike timing *m*_Δ*t*_ = −15 ms and eCB-tLTD (blue full line) with 100 pairings of *m*_Δ*t*_ = +20 ms. NMDAR-tLTP (red dashed line) corresponded to 100 pairings of *m*_Δ*t*_ = −15 ms. eCB-tLTD and eCB-tLTP are weakly affected by the jitter. NMDAR-tLTP is less robust. Full and dashed lines show averages and light swaths show ± 1 sem. (**b**) Evolution of NMDAR-tLTP, eCB-tLTD and eCB-tLTP domains for *σ*_Δ*t*_ = 0 (**b1**), 5 (**b2**) and 10 ms (**b3**) for the STDP protocol of (**a**). Note the persistence of the eCB-tLTD domain (blue box) while eCB-tLTP (red box) and NMDAR-tLTP domain (red dashed box) disappeared for 5 and 10 ms, respectively. (**c**–**e**) Similar results are obtained with other jitter distributions, including Gaussian-distributed jitter (**c**) and triangularly-distributed jitter (**d**). We also considered a combination of triangularly-distributed jitter with random IPIs distributed according to an exponential distribution with refractory period *τ*_*r*_ = 0.95 s (see *Methods*) in (**e**). The plots of the amplitude of NMDAR-tLTP, eCB-tLTD and eCB-tLTP for increasing jitter amplitude *σ*_Δ*t*_ (**c2, d2, e2**) show similar profiles and are similar to panel (**a2)**, indicating that the model predictions are not qualitatively modified by the probability distribution function of the jitter.

In Fig. 2, the jitter *ξ*_Δ*t*_ was sampled from a uniform distribution in [−*σ*_Δ*t*_, +*σ*_Δ*t*_] (Fig. 2a1). STDP in the model was globally not robust to jitter, since the three forms of plasticity vanished when *σ*_Δ*t*_ was large enough (Fig. 2a2). However, the model delivered the prediction that the three forms of plasticity do not display the same sensitivity to jitter. In particular, NMDAR-tLTP is predicted to be very fragile when subjected to *σ*_Δ*t*_: its amplitude decreases as soon as *σ*_Δ*t*_ > 2 ms and completely vanishes for *σ*_Δ*t*_ > 4 ms (Fig. 2a2 and b2). In contrast, eCB-plasticity is much less sensitive: eCB-tLTP is still present for jitters as large as 7–8 ms and eCB-tLTD is still expressed with *σ*_Δ*t*_ = 10 ms (Fig. 2b3).

We next evaluated the validity of this prediction when the probability distribution function of the jitter changes. Figure 2 illustrates the results we obtained with Gaussian (Fig. 2c) or triangular (Fig. 2d) distributions of *σ*_Δ*t*_ (see *Methods*). In both cases, the profiles of the robustness curves (Fig. 2c2 and d2) are almost identical to each other and similar to the robustness curves obtained with a uniform distribution (Fig. 2a2). In Fig. 2e, we combined a triangular distribution for *σ*_Δ*t*_ with random IPIs, i.e. in this case, stochasticity is applied not only to the timing between the two stimulations of a given pairing, but also to the time interval between two consecutive pairings: IPI distribution was Poisson with refractory period *τ*_*r*_ and rate *λ* (see *Methods*). Despite this increased stochasticity, we found that eCB-tLTD was again more robust, tolerating jitters up to 6–7 ms (eCB-tLTP) or 10 ms (eCB-tLTD) whereas NMDAR-tLTP was fragile, vanishing for *σ*_Δ*t*_ > 4 ms (Fig. 2e2). Therefore, according to our model, the observation that eCB-STDP is more robust to jitter than NMDAR-tLTP could be a general property of the response of the signaling pathways to noisy paired stimulations.

### Differential effect of jitter on NMDAR- and eCB-mediated plasticity

**Figure 3:**
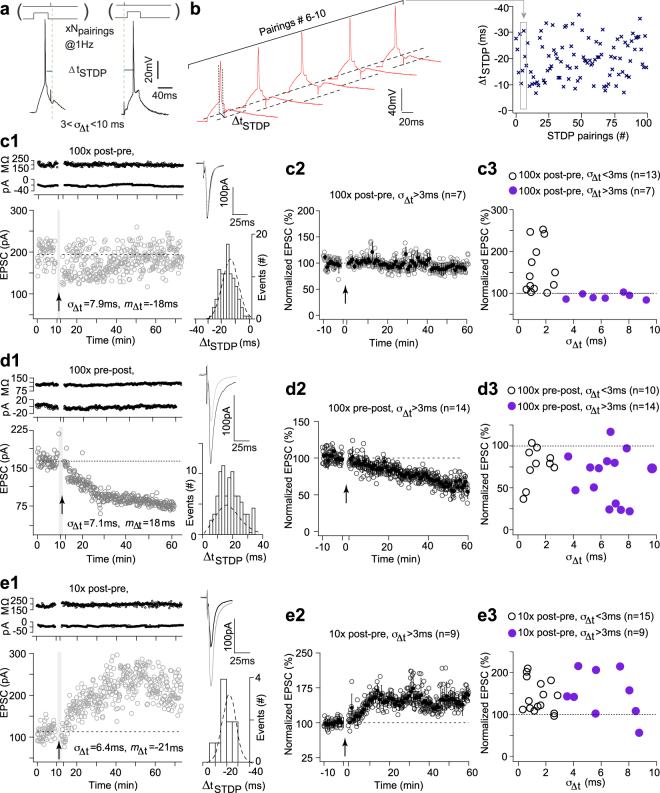
Differential effect of jitter on NMDAR- and eCB-mediated plasticity. (**a**) STDP protocol; Δ*t*_STDP_ indicates the time between pre- and postsynaptic stimulations. Δ*t*_STDP_ < 0 and Δ*t*_STDP_ > 0 refer to post-pre and pre-post pairings, respectively. (**b**) Example of 5 successive pairings (#5–9, from c1) exhibiting jittered values of Δ*t*_STDP_ (#5–9: −10.3, −15.2, −30.5, −20.7 and −17.3 ms); these pairings are indicated with the grey frame within the 100 pairings displaying jittered Δ*t*_STDP_. The distribution of the successive Δ*t*_STDP_ illustrate their standard deviation *σ*_Δ*t*_ = 7.9 ms. (**c**) NMDAR-tLTP is prevented with *σ*_Δ*t*_ > 3 ms. (**c1**) Example of the absence of plasticity after 100 post-pre pairings with *m*_Δ*t*_ = −18 ms and *σ*_Δ*t*_ = 7.9 ms; the mean baseline EPSC amplitude, 194 ± 3 pA, did not show significant change 45 minutes after pairing, 186 ± 4 pA. Upper panel, time course of Ri (baseline: 198 ± 2 MΩ and 45–55 min after pairings: 194 ± 2 MΩ; change of 2%) and holding current (I_hold_). Inset: distribution of the 100 Δ*t*_STDP_. (**c2**) Averaged time-courses of the absence of plasticity after 100 post-pre pairings with 3 < *σ*_Δ*t*_ < 10 ms. (**c3**) Relationship between the STDP magnitude and the jitter. (**d**) eCB-tLTD is not affected by 3 < *σ*_Δ*t*_ < 10 ms. (**d1**) Example of tLTD induced by 100 pre-post pairings with *m*_Δ*t*_ = +18 ms and *σ*_Δ*t*_ = 7.1 ms; the mean baseline EPSC amplitude, 160 ± 3 pA, decreased by 50%, to 81 ± 5 pA, 45 minutes after pairings. Upper panel, time course of Ri (baseline: 112 ± 1 MΩ and 35–45 min after pairings: 118 ± 1 MΩ; change of 5%) and I_hold_. Inset: distribution of the 100 Δ*t*_STDP_. (**d2**) Averaged time-courses of tLTD induced by 100 pre-post pairings with 3 < *σ*_Δ*t*_ < 10 ms. (**d3**) Relationship between the STDP magnitude and the jitter. (**e**) eCB-tLTP is not affected by 3 < *σ*_Δ*t*_ < 10 ms. (**e1**) Example of tLTP induced by 10 post-pre pairings with *m*_Δ*t*_ = −21 ms and *σ*_Δ*t*_ = 6.4 ms; the mean baseline EPSC amplitude, 113 ± 2 pA, increased by 115% to 243 ± 6 pA 45 minutes after pairings. Upper panel, time course of Ri (baseline: 185 ± 1 MΩ and 45–55 min after pairings: 197 ± 2 MΩ; change of 6%) and I_hold_. Inset: distribution of the 10 Δ*t*_STDP_. (**e2**) Averaged time-courses of tLTP induced by 10 post-pre pairings with 3 < *σ*_Δ*t*_ < 10 ms. (**e3**) Relationship between the STDP magnitude and the jitter. Insets: average of 12 successive EPSC amplitude at baseline (black trace) and at 40–50 min after STDP pairings (grey trace). Error bars represent the SEM.

Based on the model predictions, we next investigated experimentally the sensitivity to jitter of NMDAR-tLTP induced by 100 post-pre pairings (Fig. 3a), using stochastic Δ*t*_STDP_, distributed according to Fig. 2c1. As aforementioned, NMDAR-tLTP can be induced with a *σ*_Δ*t*_ < 3 ms (Fig. 1c3). Increasing *σ*_Δ*t*_ beyond 3 ms was sufficient to obliterate tLTP for 100 post-pre pairings. As exemplified (Fig. 3b and c1), 100 post-pre pairings with *σ*_Δ*t*_ = 7.9 ms and centered on *m*_Δ*t*_ = −18 ms failed to induce plasticity. Overall, we did not observe tLTP expression for *σ*_Δ*t*_ > 3 ms (92 ± 2%, *p* = 0.560, n = 7; *p* < 0.0001 when compared with tLTP observed without jitter) (Fig. 3c2 and c3) in agreement with the model prediction (Fig. 2c2).

We next investigated the robustness of the eCB-tLTD to the variability of Δ*t*_STDP_. We observed potent tLTD even with large values of *σ*_Δ*t*_. As shown in Fig. 3d1, 100 pre-post pairings with *σ*_Δ*t*_ = 7.0 ms centered on *m*_Δ*t*_ = +18 ms induced a large tLTD. Overall, we observed that tLTD was still induced for 100 pre-post pairings with 3 < *σ*_Δ*t*_ < 10 ms (70 ± 6%, *p* < 0.0001, n = 14; *p* = 0.1661 when compared with tLTD observed without jitter) (Fig. 3d2 and d3). The mean value of tLTD, observed for 3 < *σ*_Δ*t*_ < 10 ms, was not different from the one observed in control, i.e. for 0 < *σ*_Δ*t*_ < 3 ms (*p* = 0.400).

Finally, we investigated the robustness of the eCB-tLTP to the variability of Δ*t*_STDP_ and observed potent tLTP even with large values of jitter, i.e. up to *σ*_Δ*t*_ ~ 8 ms. An example of tLTP induced by 10 post-pre pairings with *σ*_Δ*t*_ = 6.4 ms centered on *m*_Δ*t*_ = −21 ms is illustrated in Fig. 3e1. In summary, tLTP could be induced even for *σ*_Δ*t*_ = 8 ms (160 ± 12%, p = 0.0021, n = 9; *p* = 0.0732 when compared with tLTP observed without jitter) (Fig. 3e2 and e3); the mean value of tLTP was not different from the one observed in control (*p* = 0.1660).

We ensured that the *m*_Δ*t*_ absolute values were not different in control and jittered conditions for 100 post-pre pairings (18 ± 2 ms, n = 13, *vs* 21 ± 3 ms, n = 7, p = 0.4711), 100 pre-post pairings (21 ± 2 ms, n = 10, *vs* 21 ± 2 ms, n = 14, p = 0.8984), 10 post-pre pairings (17 ± 2 ms, n = 15, *vs* 20 ± 2 ms, n = 9, p = 0.2297) or between these different STDP protocols (one-way ANOVA: p = 0.4851) (Supplementary Fig. 1).

In conclusion, in agreement with our model prediction, whereas NMDAR-tLTP appears fragile against the variability of Δ*t*_STDP_, eCB-plasticity (eCB-tLTD as well as eCB-tLTP) exhibits a large robustness for the temporal imprecision of Δ*t*_STDP_.

### Model-based analysis of the effects of jittering

The above experimental results provide us with a validation of our mathematical model. We next used this validated model as a tool to investigate the molecular mechanisms behind these differences of robustness.

**Figure 4:**
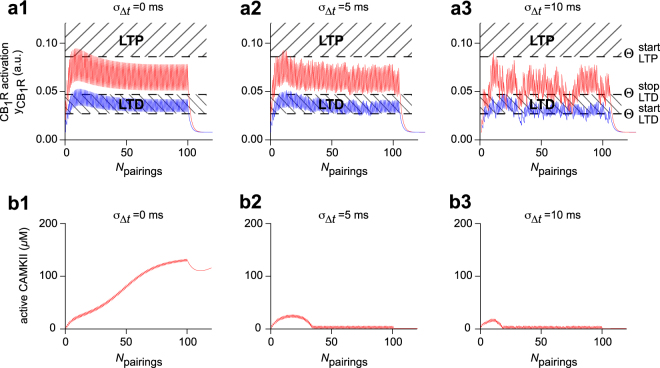
Model-based analysis of the effects of jittering. Model-predicted time-courses of (**a**) CB_1_R activation *y*_CB1R_ and (**b**) the concentration of active CaMKII during a protocol with jitter amplitude *σ*_Δ*t*_ = 0 (**a1**,**b1**), 5 (**a2**,**b2**) or 1 0 (**a3**,**b3**) ms. The STDP protocol was the same as Fig. 2a1 *i.e*. uniformly-distributed jitter of the spike timing and deterministic IPIs at 1 Hz. eCB-tLTP (**a**, red full line) and NMDAR-tLTP (**b**, red dashed line) were triggered with average spike timing *m*_Δ*t*_ = −15 ms and eCB-tLTD (**a**, blue full line) with *m*_Δ*t*_ = +20 ms. In (**a**) the shaded boxes locate the areas where *y*_CB1R_ triggers eCB-tLTP or eCB-tLTD. Those areas are defined by the thresholds ${\theta }_{{\rm{LTP}}}^{{\rm{start}}}\,\,$and (${\theta }_{{\rm{LTD}}}^{{\rm{start}}}$, ${\theta }_{{\rm{LTD}}}^{{\rm{stop}}}$), respectively (see *Methods*). For *m*_Δ*t*_ = −15 ms (red), increasing jitter amplitudes progressively hinders the build up of the *y*_CB1R_ transients. *y*_CB1R_ still reaches the LTP area for 5–25 pairings with *σ*_Δ*t*_ = 0 (**a1**) and 5 ms (**a2**) thus triggering eCB-tLTP but fails to do so for *σ*_Δ*t*_ = 10 ms (**a3**). Conversely, eCB-tLTD is robust to jitter because *y*_CB1R_ remains in the LTD region for most of the pairings with *m*_Δ*t*_ = +20 ms (blue), for all tested jitter amplitudes. In (**b**), with *m*_Δ*t*_ = −15 ms (red) the switch from the low activation to the high activation state of CaMKII is obtained in the absence of jittering *σ*_Δ*t*_ = 0 ms (**b1**). The progressive build up of activated CaMKII is efficiently suppressed as soon as *σ*_Δ*t*_ = 5 ms (**b2**) thus effectively suppressing NMDAR-tLTP (**b3**).

We have shown in a previous study that eCB-tLTP was expressed when the amount of eCBs produced was large enough that the fraction of activated CB_1_R, *y*_CB1R_, overcomes a threshold ${\theta }_{{\rm{LTP}}}^{{\rm{start}}}$^13^. In the absence of added noise, *σ*_Δ*t*_ = 0, our experimental data shows that as few as 5 pairings at 1 Hz and Δ*t*_STDP_ = −15 ms are enough to trigger eCB-tLTP^12^. Accordingly, our model produces large amounts of eCBs in the first 5–10 pre-post pairings so that *y*_CB1R_ reaches ${\theta }_{{\rm{LTP}}}^{{\rm{start}}}$ after as few as 5 pairings (Fig. 4a1). When *σ*_Δ*t*_ = 5 ms jitter was added to *m*_Δ*t*_ = −15 ms, some of the pairings failed to deliver *y*_CB1R_ transients of maximal amplitude (Fig. 4a2). But for *σ*_Δ*t*_ < 7–8 ms, a number of *y*_CB1R_ transients still had sufficient amplitude to overcome ${\theta }_{{\rm{LTP}}}^{{\rm{start}}}$, which was enough to trigger eCB-tLTP (Fig. 4a2). When *σ*_Δ*t*_ was large though (e.g. 10 ms), the *y*_CB1R_ transients failed to reach the LTP zone and eCB-tLTP was not expressed (Fig. 4a3).

In experiments without added jitter, eCB-tLTD progressively accumulates when *N*_pairings_ increases and starts to be significant for *N*_pairings_ > 25^12^. In the model with *m*_Δ*t*_ = +20 ms and *σ*_Δ*t*_ = 0, the *y*_CB1R_ transients remain in the LTD area for the most part of the STDP pairings (Fig. 4a3). To account for the progressive accumulation of eCB-tLTP with *N*_pairings_, each transient in the model contributes a small decrease of the synaptic weight. Even with large amounts of jitter (see e.g. *σ*_Δ*t*_ = 5 or 10 ms in Fig. 4a2,a3) *y*_CB1R_ transients remain mostly inside LTD area so eCB-tLTD remains expressed even with large jitter.

### The robustness of NMDAR-tLTP is predicted to be frequency-dependent

Experimentally, NMDAR-tLTP (*m*_Δ*t*_ = −15 ms and *σ*_Δ*t*_ = 0) was observed at *F*_pairings_ = 1 Hz when *N*_pairings_ > 50, beyond which its amplitude did not depend much on *N*_pairings_^13^. To account for this feature, the steady-state concentration of activated CaMKII, that sets *W*_post_ in the model, is bistable: 45 min after the stimulation, CaMKII was either almost fully inactivated (“no plasticity” state) or almost fully activated (“LTP” state)^13,38^. When the frequency of post-pre pairings (−30 < Δ*t*_STDP_ < 0 ms) was large enough (i.e. *F*_pairings_ ≥ 1 Hz), the IPI was smaller than the decay time of the CaMKII activation transient triggered by each post-pre pairings (Fig. 4b1). As a result, in the absence of added noise (*σ*_Δ*t*_ = 0), the CaMKII activation transients progressively built up on top of each other. The accumulated CaMKII activation overcomes the threshold between the “no plasticity” and the “LTP” states only for *N*_pairings_ > 50 (Fig. 4b1)^13^. With jitter (e.g. *σ*_Δ*t*_ = 5 ms in Fig. 4b2 or 10 ms in Fig. 4b3), many of the IPIs were either too long or too short to trigger maximal amplitude transients of activated CaMKII. As a result, CaMKII activation never reached the threshold, thus explaining the fragility of NMDAR-tLTP with respect to jittering (at 1 Hz).

**Figure 5:**
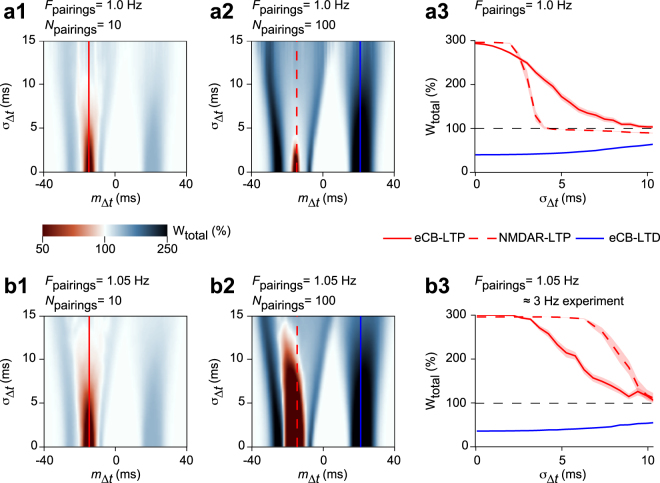
Model-based analysis of the change of robustness with frequency. (**a**) Summary of the robustness of the model plasticity at 1 Hz. The jittered STDP protocol combined uniformly-distributed jitter of the spike timing and constant IPIs at 1 Hz. Two-dimensional maps show alterations of the plasticity obtained with 10 (**a1**) or 100 (**a2**) pairings, when the average spike timing *m*_Δ*t*_ and the jitter amplitude *σ*_Δ*t*_ are changed. The locations of the plasticity blotches are indicated with horizontal lines: full red for eCB-tLTP, full blue for eCB-tLTD and dashed red for NMDAR-tLTP. The length of each of these blotches along the y-axis reflects their robustness to jitter. (**a3**) A cross-section along the dashed and full horizontal lines of (**a1**,**a2**) illustrates the robustness of NMDAR-tLTP, eCB-tLTD and eCB-tLTP. (**b**) Robustness of the model with larger frequency (1.05 Hz) and same jittered STDP protocol as in (**a**). Note the distortion between the pairing frequency in the model and in the experiments. The behavior of the model for *F*_pairings_ = 1.05 Hz corresponds to experimental results with *F*_pairings_ ≈ 3 Hz^13^. The two-dimensional maps for 10 (**b1**) or 100 (**b2**) pairings as well as the cross-sections (**b3**) predict that for frequencies larger than 1 Hz, the robustness changes, with NMDAR-tLTP becoming more robust.

A major indication from the above analysis is the importance of the IPI frequency for the robustness of NMDAR-tLTP to jitter. Figure 5 compares the robustness to jitter for constant IPI with *F*_pairings_ = 1 Hz (Fig. 5a) and 1.05 Hz (Fig. 5b). The size of the green area that locates eCB-tLTD on Fig. 5a2,b2 was not much altered by the increase of the pairing frequency. Likewise, the size of the red area that locates eCB-tLTP in Fig. 5a1,b1 did not vary much when the frequency was increased. However, the size of the red area locating NMDAR-tLTP increased drastically with frequency (Fig. 5a2,b2). Therefore, our model predicts that NMDAR-tLTP should become more robust to jitter at larger frequencies, whereas eCB-tLTP and eCB-tLTD are not significantly changed.

Several aspects of glutamate signaling at the corticostriatal synapse are known to display complex frequency-dependence^39^. Our model is calibrated with experimental data at 1 Hz and features none of the above frequency dependencies. Therefore, we cannot expect a precise quantitative match between experiments and model when frequency is varied below or above 1 Hz. However, the model still yields correct predictions of the main qualitative trends observed in the experiments, since the effects of a small change of *F*_pairings_ in the model (1.00 to 1.05 Hz) were similar to the effects of larger changes (1 to 3 Hz) in the experiments^13^. Therefore, in the experiments, we expect to observe a change of robustness pattern similar to Fig. 5b, around *F*_pairings_ = 3 Hz rather than 1.05 Hz.

### Increasing *F*_pairings_ and *N*_pairings_ stabilized NMDAR-tLTP against jitter

**Figure 6:**
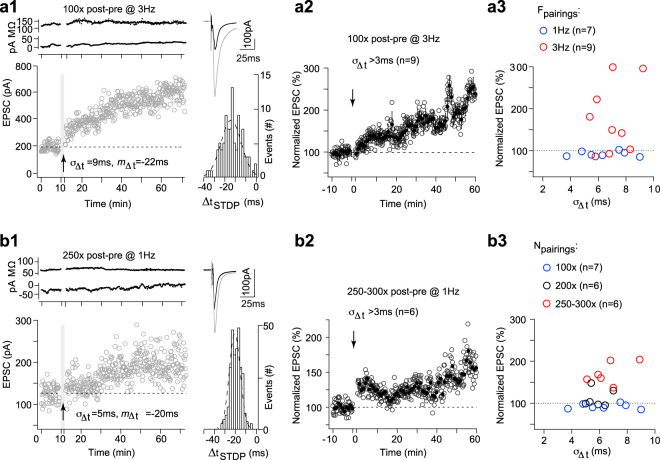
Increasing frequency and N_**pairings**_ stabilized NMDAR-tLTP against jitter. (**a**) 100 post-pre pairings at 3 Hz induced a tLTP, which is not affected by 3 < *σ*_Δ*t*_ < 10 ms. (**a1**) Example of tLTP induced by 100 post-pre pairings at 3 Hz with *m*_Δ*t*_ = −22 ms and *σ*_Δ*t*_ = 9 ms; the mean baseline EPSC amplitude, 194 ± 4 pA, increased by 212%, to 606 ± 7 pA, 45 min after pairings. Upper panel, time course of Ri (baseline: 123 ± 1 MΩ and 45–55 min after pairings: 138 ± 1 MΩ; change of 12%) and I_hold_ for this cell. Inset: distribution of Δ*t*_STDP_ for the 100 pairings. (**a2**) Averaged time-courses of tLTP induced by 100 post-pre pairings at 3 Hz with 3 < *σ*_Δ*t*_ < 10 ms. (**a3**) Relationship between the STDP magnitude and the jitter for each of the recorded neurons after 100 pairings at 1 Hz (n = 7; blue circles) or at 3 Hz (n = 10; red circles). (**b**) 250–300 post-pre pairings induced tLTP, which is not affected by 3 < *σ*_Δ*t*_ < 10 ms. (**b1**) Example of tLTP induced by 250 post-pre pairings with *m*_Δ*t*_ = −20 ms and *σ*_Δ*t*_ = 5 ms; the mean baseline EPSC amplitude, 126 ± 3 pA, increased by 57% to 198 ± 4 pA 45 minutes after pairings. Upper panel, time course of Ri (baseline: 65 ± 1 MΩ and 45–55 min after pairings: 65 ± 2 MΩ; change < 1%) and I_hold_. Inset: distribution of Δ*t*_STDP_ for the 250 pairings. (**b2**) Averaged time-courses of tLTP induced by 250–300 post-pre pairings with 3 < *σ*_Δ*t*_ < 10 ms. (**b3**) Relationship between the STDP magnitude and the jitter for each of the recorded neurons after 100 (n = 7; blue circles), 200 (n = 6; black circles) or 250–300 (n = 7; red circles) post-pre pairings. Insets: average of 12 successive EPSC amplitude at baseline (black trace) and at 40–50 min after STDP pairings (grey trace). Error bars represent the SEM.

We next tested experimentally whether an increase from 1 to 3 Hz of STDP pairings would protect the NMDAR-tLTP against jitter, as predicted by the model. As shown in Fig. 6a1, 100 post-pre pairings at 3 Hz with *σ*_Δ*t*_ = 9 ms and centered on *m*_Δ*t*_ = −22 ms induced tLTP. Overall, we observed tLTP for 100 post-pre pairings applied at 3 Hz with 4 < *σ*_Δ*t*_ < 9 ms (181 ± 30%, p = 0.0274, n = 9) (Fig. 6a2 and a3); the mean value of these tLTP observed at 3 Hz with 4 < *σ*_Δ*t*_ < 9 ms were not significantly different from the one observed with stimulation-related *σ*_Δ*t*_ < 3 ms at 1 Hz (p = 0.520). When we plotted the magnitude of plasticity against *σ*_Δ*t*_ for 100 post-pre pairings at 3 Hz, we observed that tLTP was still induced even for *σ*_Δ*t*_ = 9 ms (Fig. 6a3). We observed different synaptic efficacy changes following 100 post-pre pairings at 1 Hz with and without jitter and at 3 Hz with jitter (ANOVA, p < 0.0001). The NMDAR-tLTP thus acquires certain robustness to the variability of Δ*t*_STDP_ with increasing frequency of pairings.

We next investigated whether a higher *N*_pairings_ would secure the expression of NMDAR-tLTP even with *σ*_Δ*t*_ > 3 ms (Fig. 6b). When we doubled *N*_pairings_ we did not observe expression of significant tLTP. Indeed, 200 post-pre pairings (−30 < *m*_Δ*t*_ < 0 ms with 3 < *σ*_Δ*t*_ < 10 ms) did not induce plasticity (112 ± 9%, *p* = 0.236, *n* = 6; Fig. 6b3) but when we increased *N*_pairings_ up to 250–300, tLTP was reliably observed with *σ*_Δ*t*_ > 3 ms. Figure 6b1 shows an example of tLTP induced by 250 pairings with *σ*_Δ*t*_ = 5 ms and centered on −20 ms. Overall, 250–300 post-pre STDP pairings (−30 < *m*_Δ*t*_ < 0 ms with 3 < *σ*_Δ*t*_ < 10 ms) induced tLTP (170 ± 10%, *p* = 0.0012, n = 6; Fig. 6b2 and b3); the mean values of these tLTP observed for *N*_pairings_ = 250–300 at 1 Hz with 4 < *σ*_Δ*t*_ < 9 ms were not different compared to tLTP induced with 100 post-pre pairings at 1 Hz with *σ*_Δ*t*_ < 3 ms (*p* = 0.1952). We observed different synaptic efficacy changes following 100, 200 and 250–300 post-pre pairings (ANOVA, p < 0.0001).

### Transitions from spike-timing- to frequency-dependent plasticity

We next tested with our mathematical model whether the robustness of STDP to jitter of the spike timing depends on the regularity of the spike train. In Fig. 2e, we show an example of the combination of stochastic spike timings (triangular distribution) with stochastic IPIs (Poisson distributed) with a constant and relatively large refractory period *τ*_*r*_ (see *Methods- Mathematical model, Protocol 4*). However, when *τ*_*r*_ decreases, short IPIs are more frequently sampled and the train of presynaptic stimulation becomes more irregular. Therefore, through variations of the refractory period *τ*_*r*_, the presynaptic stimulation can be progressively switched from highly irregular to regular spike trains.

**Figure 7:**
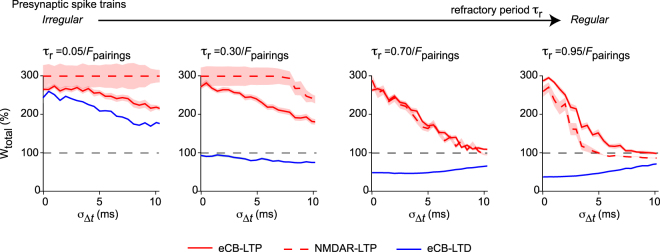
The robustness of plasticity depends on the regularity of the presynaptic spike train. The figure shows the robustness of the model plasticity (eCB-tLTP with red, eCB-tLTD with blue and NMDAR-tLTP with dashed red) with the jittered STDP protocol of Fig. 2e1, *i.e*. exponentially-distributed IPIs with refractory period *τ*_*r*_ and triangularly-distributed jitter *ξ*_Δ*t*_. From right to left, the refractory period *τ*_*r*_ is gradually decreased. When *τ*_*r*_ → 1/*F*_pairings_ where *F*_pairings_ = 1 Hz is the average pairings frequency, one gets regular presynaptic spike trains, similar to classical STDP protocols. In this case (see e.g. *τ*_*r*_ = 0.95/*F*_pairings_), the robustness pattern is similar to the previous observations at 1 Hz: eCB-STDP is robust, whereas NMDAR-tLTP is fragile. However, when *τ*_*r*_ → 0, the presynaptic spike train becomes highly irregular. In this case, the robustness patterns changes totally, with NMDAR-LTP becoming the most robust, and eCB-tLTD turning into a LTP. Hence, irregular presynaptic spike trains are predicted to produce robust LTP at the expense of LTD.

Figure 7 shows model predictions for the alteration of the robustness curves when *τ*_*r*_ varies. With high refractory period, *i.e*. when *τ*_*r*_ ≈ *F*_pairings_, the IPIs are weakly noisy, so one recovers the robustness curves of Fig. 2e, with eCB-STDP being more robust than NMDAR-tLTP. However, when *τ*_*r*_ is 70% of *F*_pairings_, the robustness of both eCB-tLTP and NMDAR-tLTP increases and both exhibit similar robustness. With even smaller values of *τ*_*r*_, NMDAR-tLTP becomes more robust than eCB-tLTP. Moreover, eCB-tLTD (*F*_pairings_ = 1 Hz, *m*_Δ*t*_ = +20 ms, *N*_pairings_ = 100) progressively stops triggering LTD when *τ*_*r*_ decreases, even inducing LTP for very low *τ*_*r*_. Hence, when the presynaptic spike train is progressively switched from regular to very irregular spike trains, two main changes occur: (*i*) the three plasticity forms (NMDAR-tLTP, eCB-tLTD and eCB-tLTP) become increasingly robust to jittering (at the limit of highly irregular spike trains, jittering hardly affects plasticity amplitudes), and (*ii*) eCB-tLTD is progressively changed to LTP, so that highly irregular paired stimulations with *F*_pairings_ = 1 Hz only produce LTP regardless of the sign of the spike timing.

## Discussion

Here, we have introduced jittered STDP protocols as noisy variants of STDP protocols where the spike timing, *i.e*. the delay between a presynaptic and paired postsynaptic stimulation (or vice-versa), is a random variable. Our results show that the magnitude of variability that STDP can tolerate varies depending on the pairing frequency and on the STDP form, i.e. NMDAR-tLTP, eCB-tLTP or eCB-tLTD. At 1 Hz pairing frequency, eCB-STDP (eCB-tLTD and eCB-tLTP) is much more robust than NMDAR-dependent tLTP. However, this robustness depends on the number and frequency of pairings. Indeed, increasing the average pairing frequency to 3 Hz improved the robustness of NMDAR-tLTP. We observed a similar improvement of the robustness of NMDAR-tLTP to jitter when the number of pairings increased more than two folds.

These results have wide-ranging implications for STDP expression in *in vivo*-like firing. They indicate that eCB-STDP could be more likely responsible for fast learning involving few trials (eCB-tLTP) or for learning at lower activity frequency than NMDAR-STDP. NMDAR-STDP could however take part in learning for larger frequencies or when the same spike timing recurs a large number of times during sustained activity. It also implies that in synapses where NMDAR is the main coincidence detector for tLTD or tLTP^3,5,6,40–42^, a high degree of precision of the spike timing, and/or an increased number or frequency of pairings, are required for the emergence of the NMDAR-mediated plasticity. This can be viewed as a mechanism preventing the occurrence of spurious plasticity for noisy neural network activity. At synapses in which NMDAR and endocannabinoids are both involved in STDP expression^12,13,25–27,33,43^ and in which eCB-LTP can occur, as it is the case in the striatum^12,13^, the hippocampus^44–48^ or the neocortex^49^ the emergence of STDP would be possible even in noisy conditions and could thus serve subsequently as a priming for the subsequent expression of NMDAR-tLTP.

In the present study, we explored the robustness of STDP when noise affects not only the spike timing but also the inter-pairing interval (IPI), i.e. the delay between two successive pairings. In the absence of jitter of the spike timing, our model predicts that IPI irregularity tends to consolidate tLTP at the expense of tLTD. This prediction that irregular IPIs strengthen tLTP when the spike timing is constant confirms the result obtained independently and with different mathematical models^7,19,22,24^. When IPIs are regular, adding jitter of the spike timing in our model progressively suppresses STDP. However, with more irregular IPIs our model predicts that the three STDP forms should exhibit larger robustness to noise. Therefore our result suggests that jittering of the spike timing has larger impact on STDP when the IPIs are regular, but that jittering has much less consequence with irregular IPIs. Experimental testing of this model prediction would greatly improve our understanding of the induction and maintenance of STDP in *in vivo-*like firing.

Subthreshold postsynaptic depolarization is a key factor in the induction of plasticity^50^. The existence of plasticity induced by STDP**-**like protocols without postsynaptic spikes (subthreshold-depolarization-dependent plasticity, SDDP) has been reported in the neocortex^51^, striatum^52^ and hippocampus^53^. In the striatum, SDDP differs from STDP on two aspects: (*i*) SDDP was induced in a larger temporal window (−100 < Δ*t*_STDP_ < +100 ms) and (*ii*) tLTP and tLTD were induced regardless of the spike timing. Therefore, the action potential would not be necessary for plasticity induction, but determinant for the STDP polarity and the width of the Δ*t*_STDP_. The effects of jitter remain to be determined on SDDP, *i.e*. subthreshold events.

The polarity of plasticity depends greatly on the frequency of stimulus presentation. Indeed, prolonged firing activity at low and high frequency promote LTD and LTP, respectively, at the same synapses in hippocampus^54–56^. However, alteration of a regular low-frequency stimulation (900 stimulations at 1 Hz) with Poisson-distributed interstimulus intervals (as an approximation of naturalistic patterns) prevents LTD expression in the neocortex^57^. Interestingly, this LTD induced with regular low-frequency stimulation has been reported to be NMDAR-mediated^58^, and we show here that NMDAR-plasticity is fragile to jitter especially for low firing rate (1 Hz). It remains thus to investigate in a rate-based paradigm whether Poisson stimulation would erase LTD induced by higher firing rate (3–5 Hz^54^). Various forms of naturalistic patterns as an attempt to mimic *in vivo* activity have been experimentally tested for plasticity expression: from single burst of dendritic spikes, which induced NMDAR-LTP in hippocampus^59^ and NMDAR-LTD in neocortex^60^, to noisy STDP-like protocols^7,19,20,22^ and burst-timing-dependent plasticity paradigms^61^. Based on data harvested in the neocortex, a phenomenological computer modeling showed that for constant frequencies but random spiking, LTP wins over LTD with increasing frequency^7^. Activity patterns recorded *in vivo* during specific behavioral tasks have been replayed *ex vivo* to test their ability to induce plasticity^19,56,62^. When sequences of spike trains recorded *in vivo* from visual cortex of the anaesthetized cat in response to natural scenes were replayed *ex vivo* in layer 2/3 pyramidal cells of rat visual cortex, both LTP and LTD was observed experimentally and modeling suggested the importance of suppressive interactions between spikes^19^. In the dentate gyrus, *in vivo* patterns recorded in granule cell during a task of delayed nonmatch-to-sample were able in most of the cases to induce LTP at mossy fibers^56^. Similarly, when hippocampal place cells activity recorded *in vivo* were replayed *ex vivo*, NMDAR-LTP (also dependent on cholinergic tone) was observed^62^. Natural stimuli recruit various neuronal circuits leading to presynaptic jitter, which can have important physiological consequences. Indeed, *in vivo* visually driven presynaptic inputs induce presynaptic jitter leading to broader membrane potential events, and can consequently promote a spatial reorganization of the responses of neurons to neighboring stimuli within a receptive field, as demonstrated in the rat visual cortex^22^.

The debate about the nature of the neural coding, i.e. rate versus time codes^63–65^, is prominent when studying STDP. It remains to be tested with mathematical models and experiments whether activity patterns of various vigilance states differently result in the emergence of spike- or rate-coding plasticity. At most of the synapses both rate and time codes co-exist; for example at CA1 synapses, stimuli induced LTD at 2–3 Hz, no plasticity at 10 Hz and LTP at 50 Hz demonstrating a rate code^54^, and time codes are also supported as illustrated by the expression of various forms of STDP at the same synapse^11^. An answer to this debate could be that both rate and time codes work together for plasticity expression, as recently shown in the somatosensory cortex^66^, but their relative contribution *in vivo* would depend on the ongoing activity (low or high), the cerebral structures, the noise level, the neuromodulatory systems at play and the signaling pathways involved in plasticity expression (as shown in the present study). Briefly, it appears that STDP can emerge form low frequency activity whereas rate codes would take over for higher frequency ranges^7,19,20,22^.

A major conclusion from our present work is that the robustness of STDP to jitter depends on the underlying signaling pathways. For instance, the robustness of NMDAR-tLTP in our model is dependent on the amplitude of the activated CaMKII transients triggered by each post-pre pairings or on alterations of the ratio between the decay time of these transients and the IPIs. The robustness of STDP to jitter could be similarly controlled by quantitative variations in the underlying pathways. Such variations are expected to occur between two neuronal subtypes or brain regions but also as a result of the activation of a neuromodulatory pathway. Therefore, our work suggests that the expression of STDP in *in vivo*-like firing might appear or disappear as a result of modulations of its robustness to jitter, depending on the properties of the incoming patterns (reflecting for example fast learning or heavy training), but also on the brain region and, more transiently, on the activation of neuromodulatory pathways.

## Methods

### Animals and brain slice preparation

OFA (Oncins France Strain A) rats (Charles River, L’Arbresle, France) were used at postnatal day 25–32 for brain slice electrophysiology. All experiments were performed in accordance with the guidelines of the local animal welfare committee (Center for Interdisciplinary Research in Biology Ethics Committee) and the EU (directive 2010/63/EU). The experimental protocol (ref. 2017–02 N° 2016120110255176) was approved by the Ethics Committee in Charge of Animal Experimentation (Paris Centre et Sud). Every precaution was taken to minimize stress and the number of animals used in each series of experiments. Animals were housed in standard 12 hours light/dark cycles and food and water were available *ad libitum*. Horizontal brain slices containing the somatosensory cortical area and the corresponding corticostriatal projection field were prepared as previously described^36^. Corticostriatal connections (between somatosensory cortex layer 5 and the dorso-lateral striatum) are preserved in the horizontal plane. Brain slices (330 µm-thick) were prepared with a vibrating blade microtome (VT1200S, Leica Microsystems, Nussloch, Germany). Brains were sliced in an ice-cold cutting solution (125 mM NaCl, 2.5 mM KCl, 25 mM glucose 25 mM NaHCO_3_, 1.25 mM NaH_2_PO_4_, 2 mM CaCl_2_, 1 mM MgCl_2_, 1 mM pyruvic acid) through which 95% O_2_/5% CO_2_ was bubbled. The slices were transferred to the same solution at 34 °C for one hour and then to room temperature.

### Patch-clamp recordings

Patch-clamp recordings were performed as previously described^12,18^. Briefly, for whole-cell recordings in borosilicate glass pipettes of 5–7 MΩ resistance were filled with (in mM): 122 K-gluconate, 13 KCl, 10 HEPES, 10 phosphocreatine, 4 Mg-ATP, 0.3 Na-GTP, 0.3 EGTA (adjusted to pH 7.35 with KOH). The composition of the extracellular solution was (mM): 125 NaCl, 2.5 KCl, 25 glucose, 25 NaHCO_3_, 1.25 NaH_2_PO_4_, 2 CaCl_2_, 1 MgCl_2_, 10 μM pyruvic acid bubbled with 95% O_2_ and 5% CO_2_. Signals were amplified using EPC10–2 amplifiers (HEKA Elektronik, Lambrecht, Germany). All recordings were performed at 34 °C (Bath-controller V, Luigs&Neumann, Ratingen, Germany) and slices were continuously superfused with extracellular solution, at a rate of 2 ml/min. Slices were visualized under microscope (BX51WI Olympus, Rungis, France), with a 4x/0.13 objective for the placement of the stimulating electrode and a 40x/0.80 water-immersion objective for the localization of cells for whole-cell recordings. Current- and voltage-clamp recordings were filtered at 5 kHz and sampled at 10 kHz, with the Patchmaster v2 × 32 program (HEKA Elektronik).

### Spike timing-dependent plasticity protocols: regular and jittered patterns

Electrical stimulations were performed with a concentric bipolar electrode (Phymep, Paris, France) placed in layer 5 of the somatosensory cortex. Electrical stimulation was monophasic, at constant current (ISO-Flex stimulator, AMPI, Jerusalem, Israel). Cortical stimulations evoked glutamatergic excitatory postsynaptic currents (EPSCs) (inhibited by CNQX 10 μM and D-AP5 50 μM, n = 6) and not significantly affected by GABAergic events; Indeed, blocking GABA_A_Rs with picrotoxin (50 μM) did not significantly affect EPSC amplitude at corticostriatal synapses (123 ± 31 pA before and 118 ± 26 pA after picrotoxin, *p* = 0.500, n = 5). MSNs were kept at their resting membrane potential (−74.6 ± 0.6 mV, n = 90) and repetitive control stimuli were applied at 0.1 Hz. Currents were adjusted to evoke 50–200 pA EPSCs. STDP protocols consisted of pairings of pre- and postsynaptic stimulations (at 1 Hz) separated by a specific time interval (Δ*t*_STDP_). The paired stimulations were applied at 1 Hz throughout the study except in Fig. 6a1 and a2 in which 3 Hz stimulations were tested. Presynaptic stimulations corresponded to cortical stimulations and the postsynaptic stimulation of an action potential evoked by a depolarizing current step (30 ms duration) in MSNs. Δ*t*_STDP_ < 0 ms for post-before-pre pairings, and Δ*t*_STDP_ > 0 ms for pre-before-post pairings. Neuronal recordings on neurons were made over a period of 10 minutes at baseline, and for at least 60 minutes after the STDP protocols. EPSC baseline stability was assessed by comparing the average of the EPSC magnitude of the two first and the two last minutes of baseline; if the variation of amplitude exceeded 10%, the experiment was discarded^4^. Recordings were stopped if the injected current was larger than 50 or 100–150 pA during the baseline and after pairings, respectively. We individually measured and averaged the amplitude of 60 successive EPSCs from both baseline and 45–55 minutes after STDP protocol, in which the latter was normalized by the former to calculate long-term synaptic efficacy changes. Neuron recordings were made in voltage-clamp mode during baseline and for the 60 minutes of recording after the STDP protocol, and in current-clamp mode during STDP protocol. Experiments were excluded if the input resistance (Ri), measured every 10 sec all along the experiment, varied by more than 20% during the very same time period where the EPSC amplitude were measured for plasticity assessment: 10 minutes of baseline and the 10 last minutes of the recording (generally 45–55 minutes after pairings). After recording of 10 min control baseline, drugs were applied in the bath. A new baseline with drugs was recorded after a time lapse of 10 min (to allow the drug to be fully perfused) for 10 min before the STDP protocol. Drugs were present until the end of the recording. All chemicals were purchased from Tocris (Ellisville, MO, USA), except for picrotoxin (Sigma). DL-2-amino-5-phosphono-pentanoic acid (D-AP5; 50 μM) and 6-cyano-7-nitroquinoxaline-2,3-dione (CNQX; 10 μM) were dissolved directly in the extracellular solution and bath applied. N-(piperidin-1-yl)-5-(4-iodophenyl)-1-(2,4-dichlorophenyl)-4-methyl-1H-pyrazole-3-carboxamide (AM251; 3 μM) and picrotoxin (50 μM) were dissolved in ethanol and added to the external solution, such that the final concentration of ethanol was 0.01–0.1%.

For the jittered Δ*t*_STDP_ patterns, we used the following algorithm (see Protocol 2, below). We note ${t}_{{{\rm{pre}}}_{i}}$ and ${t}_{{{\rm{post}}}_{i}}$ the times of the presynaptic and postsynaptic stimulations in the paired stimulation number *i*, respectively. To set them, we first fixed the times of each presynaptic stimulation using the pairing frequency, i.e. ${t}_{{{\rm{pre}}}_{i}}=\frac{i}{{F}_{{\rm{pairings}}}}$. The postsynaptic times were then chosen randomly according to ${t}_{{{\rm{post}}}_{i}}$ = ${t}_{{{\rm{pre}}}_{i}}$ + *m*_Δ*t*_ + *ξ*_Δ*t*,*i*_ where *m*_Δ*t*_ is the average spike timing and *ξ*_Δ*t*_ is a random variable with mean 0 and variance ${\sigma }_{{\rm{\Delta }}t}^{2}$. We defined the Inter-Pairing-Interval (IPI) as the time interval between the presynaptic stimulation times of two consecutive paired stimulations (${{\rm{IPI}}}_{i}={t}_{{{\rm{pre}}}_{i+1}}-{t}_{{{\rm{pre}}}_{i}}$), whereas the spike timing $\xi t$ is the time interval between the postsynaptic and the presynaptic stimulation times within a given paired stimulation (${\rm{\Delta }}{t}_{{{\rm{STDP}}}_{i}}={t}_{{{\rm{post}}}_{i}}-{t}_{{{\rm{pre}}}_{i}}$). In a standard STDP protocol both the spike timing and the IPI are constant. The above algorithm yields constant IPIs and stochastic spike timings.

### Electrophysiological data analysis

Off-line analysis was performed with Fitmaster (Heka Elektronik) and Igor-Pro 6.0.3 (Wavemetrics). Statistical analysis was performed with Prism 5.02 software. In all cases “n” refers to an experiment on a single cell from a single slice. All results are expressed as mean ± SEM in the text and as mean ± SD in the figures. Statistical significance was assessed in unpaired *t* tests, one way ANOVA, or in one-sample *t* tests, as appropriate, using the indicated significance threshold (*p*).

### Mathematical model

#### Jittered STDP protocols

In the model, we define the spike timing as ${\rm{\Delta }}{t}_{{{\rm{STDP}}}_{i}}=$ ${t}_{{{\rm{post}}}_{i}}-{t}_{{{\rm{pre}}}_{i}}+\delta$ where *δ* accounts for the time elapsed between the onset of the postsynaptic step current and the action potential it triggers (~3 ms in MSNs). In agreement with the experiment protocol above, we introduced stochasticity of the spike timing by adding a random jitter *ξ*_Δ*t*_ to the spike timing: Δ*t*_STDP_ = *m*_Δ*t*_ + *ξ*_Δ*t*_, were *m*_Δ*t*_ is the average spike timing and *ξ*_Δ*t*_ is a random variable whose distribution is given by the STDP protocol. Here, we explored five STDP protocols:

1. *Protocol 0* consisted of pairings with no jittering, i.e. ${{\rm{IPI}}}_{i}=\frac{1}{{F}_{{\rm{pairings}}}}$ and *ξ*_Δ*t*_ = 0.
2. *Protocol 1* consisted of deterministic IPIs and uniformly distributed spike timings (Fig. 2a1), i.e. ${{\rm{IPI}}}_{i}=\frac{1}{{F}_{{\rm{pairings}}}},\,\forall i$, and the probability distribution function of the jitter is given by
  1 $$P({\xi }_{{\rm{\Delta }}t})=\{\begin{array}{cc}\frac{1}{2{\sigma }_{{\rm{\Delta }}t}} & {\rm{i}}{\rm{f}}\,|{\xi }_{{\rm{\Delta }}t}| < {\sigma }_{{\rm{\Delta }}t},\\ 0 & {\rm{o}}{\rm{t}}{\rm{h}}{\rm{e}}{\rm{r}}{\rm{w}}{\rm{i}}{\rm{s}}{\rm{e}}\end{array}$$
  In eq. (1), *σ*_Δ*t*_ defines the maximal jitter amplitude *i.e*. the level of noise in the protocol. To simulate *Protocol 1*, we first set each presynaptic stimulation time using ${t}_{{{\rm{pre}}}_{i}}=\frac{i}{{F}_{{\rm{pairings}}}}$. Postsynaptic times were then fixed by ${t}_{{{\rm{p}}{\rm{o}}{\rm{s}}{\rm{t}}}_{i}}$ = ${t}_{{{\rm{pre}}}_{i}}$ + *m*_Δ*t*_ + *ξ*_Δ*t*,*i*_ − *δ*.
3. *Protocol 2* (Fig. 2c1) shared the same definition as Protocol 1, except that the jitter followed a normal distribution with zero mean and variance ${\sigma }_{{\rm{\Delta }}t}^{2}$: $P({\xi }_{{\rm{\Delta }}t})=\,{\mathscr{N}}(0,\,{\sigma }_{{\rm{\Delta }}t}^{2})$.
4. For *Protocol 3* (Fig. 2d1), we used a triangular distribution of the jitter by adding uniformly distributed jitter to both presynaptic and postsynaptic times. We set ${t}_{{{\rm{pre}}}_{i}}=\frac{i}{{F}_{{\rm{pairings}}}}+{\zeta }_{{\rm{pre}},i}$ and ${t}_{{{\rm{post}}}_{i}}=\frac{i}{{F}_{{\rm{pairings}}}}+{m}_{{\rm{\Delta }}t}+{\zeta }_{{\rm{post}},i}-\delta$, where *ζ*_pre_ and *ζ*_post_ are i.i.d. random variables with the uniform distribution of eq. (1). The resulting spike timing is Δ*t*_STDP_ = *m*_Δ*t*_ + *ξ*_Δ*t*_ where *ξ*_Δ*t*_ = *ζ*_post_ − *ζ*_pre_ has a triangular distribution. Note that in this case, one still has ${{\rm{IPI}}}_{i}\approx \frac{1}{{F}_{{\rm{pairings}}}}\,\forall i$ as long as 2|*m*_Δ*t*_|*F*_pairings_ ≪ 1, which can safely be assumed here since we used |*m*_Δ*t*_| < 50 ms and *F*_pairings_ < 2 Hz.
5. *Protocol 4* includes both a stochastic spike timing and stochastic IPIs (Fig. 2e1). We first sampled *N*_pairings_ IPIs according to an exponential distribution with rate *λ* and refractory period *τ*_*r*_: *P*(IPI) = Θ (IPI − *τ*_*r*_)*λ* exp (−*λ*(IPI − *τ*_*r*_)), where Θ(*x*) is the Heaviside function Θ (*x*) = 1 if *x* ≥ 0; 0 otherwise. The spike trains defined with protocol 4 are thus Poisson process. We used these IPIs to fix the stimulation times, adding a triangularly distributed jitter as in Protocol 3 above: ${t}_{{{\rm{pre}}}_{i}}={\sum }_{j < i}{{\rm{IPI}}}_{j}+{\zeta }_{{\rm{pre}},i}$ and ${t}_{{{\rm{post}}}_{i}}={\sum }_{j < i}{{\rm{IPI}}}_{j}+{m}_{{\rm{\Delta }}t}+{\zeta }_{{\rm{post}},i}-\delta$. To keep the average stimulation frequency at 1/*F*_pairings_, we constrained the value of the rate *λ* as *λ* = (1/*F*_pairings_ − *τ*_*r*_)^−1^.

#### Stimulations

A detailed account of our mathematical model can be found in S1 Text. We modeled glutamate concentration in the synaptic cleft, *G*(*t*), as a train of exponentially-decaying impulses triggered by presynaptic stimuli at time ${t}_{{{\rm{pre}}}_{i}}$. We modeled the electrical response to these stimulations in a postsynaptic element considered as a single isopotential compartment with AMPAR, NMDAR, VSCC and TRPV1 conductances:

2 $$\begin{array}{rcl}{C}_{{\rm{m}}}dV/dt & = & -{g}_{L}(V-{V}_{{\rm{L}}})-{I}_{{\rm{AMPAR}}}(V,G(t))-{I}_{{\rm{NMDAR}}}(V,G(t))\\  &  & -{I}_{{\rm{VSCC}}}(V)-{I}_{{\rm{TRPV}}1}(V,AEA)-{I}_{{\rm{action}}}(t)\end{array}$$

where *V* is membrane potential; *g*_L_ and *V*_L_ are leak conductance and reversal potential respectively; *I*_AMPAR_, *I*_NMDAR_, *I*_VSCC_ and *I*_TRPV1_ are currents through AMPAR, NMDAR, L-type VSCC (v1.3) and TRPV1 respectively. *I*_action_ is the action current resulting from the postsynaptic stimulation (backpropagating action potential on top of a 30 ms depolarization). Details about the analytical expressions of these currents are given in S1 Text.

#### Biochemical signaling

We modeled the kinetics of the biochemical pathways activated by the above electrical stimulations using the model of ref.^13^. We give below a quick overview of this model and refer to S1 Text for the details. Free cytosolic calcium is one of the main signaling actors in the model. To model its dynamics, we assumed calcium can be transferred from/to two main sources: (*i*) extracellular calcium, via the plasma membrane channels of eq. (2) above and (*ii*) the endoplasmic reticulum (ER), via the IP3-dependent calcium-induced calcium release system. These calcium transients activated a network of biochemical pathways that collectively set the synaptic weight. Hence, in this model, the synaptic weight is entirely fixed by the underlying biochemical signaling network^13^. More precisely, we assumed that the total synaptic weight is the product of a pre- and a postsynaptic contribution *W*_total_ = *W*_pre_*W*_post_. Postsynaptic plasticity was based on the activation by calcium of calmodulin and CaMKII^67,68^. We assumed that postsynaptic plasticity is directly proportional to the calcium-dependent activation of CaMKII:

3 $${W}_{{\rm{post}}}=1+3.5\frac{CaMKI{I}^{\ast }}{CaMKI{I}_{{\rm{\max }}}^{\ast }}$$

where CaMKII^*^ and $CaMKI{I}_{{\rm{\max }}}^{\ast }$ are the current concentration of activated (phosphorylated) CaMKII and its maximum value.

Our model also accounts for the biochemical pathways leading to the production of the endocannabinoids 2-arachidonoylglycerol (2-AG) and AEA, and their subsequent activation of CB_1_R (see S1 Text for further details). In our model, CB_1_R activation (*y*_CB1R_) controls the presynaptic weight *W*_pre_ according to the following rule:

4 $${\rm{\Omega }}({y}_{{\rm{C}}{\rm{B}}1{\rm{R}}})=\{\begin{array}{cc}1-{A}_{{\rm{L}}{\rm{T}}{\rm{D}}} & {\rm{i}}{\rm{f}}\,{{\rm{\Theta }}}_{{\rm{L}}{\rm{T}}{\rm{D}}}^{{\rm{s}}{\rm{t}}{\rm{a}}{\rm{r}}{\rm{t}}} < {y}_{{\rm{C}}{\rm{B}}1{\rm{R}}} < {{\rm{\Theta }}}_{{\rm{L}}{\rm{T}}{\rm{D}}}^{{\rm{s}}{\rm{t}}{\rm{o}}{\rm{p}}}\\ 1+{A}_{{\rm{L}}{\rm{T}}{\rm{P}}} & {\rm{i}}{\rm{f}}\,{{\rm{\Theta }}}_{{\rm{L}}{\rm{T}}{\rm{P}}}^{{\rm{s}}{\rm{t}}{\rm{a}}{\rm{r}}{\rm{t}}} < {y}_{{\rm{C}}{\rm{B}}1{\rm{R}}}\\ 1 & {\rm{o}}{\rm{t}}{\rm{h}}{\rm{e}}{\rm{r}}{\rm{w}}{\rm{i}}{\rm{s}}{\rm{e}}\end{array}$$

and

5 $${\rm{d}}{W}_{{\rm{pre}}}/dt=(\Omega ({y}_{{\rm{CB1R}}})-{W}_{{\rm{pre}}})/{\tau }_{{W}_{{\rm{pre}}}}$$

Details about model implementation and numerics are given in the supplementary information.

## Electronic supplementary material

Supporting Information

## References

[1] Takeuchi T, Duszkiewicz AJ, Morris RGM (2014). The synaptic plasticity and memory hypothesis: encoding, storage and persistence. Philos. Trans. R. Soc. Lond., B, Biol. Sci..

[2] Debanne D, Gähwiler BH, Thompson SM (1997). Bidirectional associative plasticity of unitary CA3-CA1 EPSPs in the rat hippocampus *in vitro*. J. Neurophysiol..

[3] Magee JC, Johnston D (1997). A synaptically controlled, associative signal for Hebbian plasticity in hippocampal neurons. Science.

[4] Markram H, Lübke J, Frotscher M, Sakmann B (1997). Regulation of synaptic efficacy by coincidence of postsynaptic APs and EPSPs. Science.

[5] Bi G, Poo M (1998). Synaptic Modifications in Cultured Hippocampal Neurons: Dependence on Spike Timing, Synaptic Strength, and Postsynaptic Cell Type. J. Neurosci..

[6] Zhang LI, Tao HW, Holt CE, Harris WA, Poo M (1998). A critical window for cooperation and competition among developing retinotectal synapses. Nature.

[7] Sjöström PJ, Turrigiano GG, Nelson SB (2001). Rate, timing, and cooperativity jointly determine cortical synaptic plasticity. Neuron.

[8] Feldman DE (2012). The spike-timing dependence of plasticity. Neuron.

[9] Madadi Asl M, Valizadeh A, Tass PA (2017). Dendritic and Axonal Propagation Delays Determine Emergent Structures of Neuronal Networks with Plastic Synapses. Sci Rep.

[10] Froemke RC, Tsay IA, Raad M, Long JD, Dan Y (2006). Contribution of individual spikes in burst-induced long-term synaptic modification. J. Neurophysiol..

[11] Wittenberg GM, Wang SS-H (2006). Malleability of spike-timing-dependent plasticity at the CA3-CA1 synapse. J. Neurosci..

[12] Cui Y (2015). Endocannabinoids mediate bidirectional striatal spike-timing-dependent plasticity. J. Physiol. (Lond.).

[13] Cui Y (2016). Endocannabinoid dynamics gate spike-timing dependent depression and potentiation. Elife.

[14] Sjöström PJ, Häusser M (2006). A Cooperative Switch Determines the Sign of Synaptic Plasticity in Distal Dendrites of Neocortical Pyramidal Neurons. Neuron.

[15] Clopath C, Büsing L, Vasilaki E, Gerstner W (2010). Connectivity reflects coding: a model of voltage-based STDP with homeostasis. Nat. Neurosci..

[16] Pawlak, V., Wickens, J. R., Kirkwood, A. & Kerr, J. N. D. Timing is not Everything: Neuromodulation Opens the STDP Gate. *Front Synaptic Neurosci* **2** (2010). 10.3389/fnsyn.2010.00146 PMC3059689 21423532

[17] Frémaux N, Gerstner W (2015). Neuromodulated Spike-Timing-Dependent Plasticity, and Theory of Three-Factor Learning Rules. Front Neural Circuits.

[18] Valtcheva, S. & Venance, L. Astrocytes gate Hebbian synaptic plasticity in the striatum. *Nat Commun* **7** (2016). 10.1038/ncomms13845 PMC5187441 27996006

[19] Froemke RC, Dan Y (2002). Spike-timing-dependent synaptic modification induced by natural spike trains. Nature.

[20] Celikel T, Szostak VA, Feldman DE (2004). Modulation of spike timing by sensory deprivation during induction of cortical map plasticity. Nat. Neurosci..

[21] Morrison A, Diesmann M, Gerstner W (2008). Phenomenological models of synaptic plasticity based on spike timing. Biol Cybern.

[22] Pawlak V, Greenberg DS, Sprekeler H, Gerstner W, Kerr JND (2013). Changing the responses of cortical neurons from sub- to suprathreshold using single spikes *in vivo*. Elife.

[23] Abbott LF, Nelson SB (2000). Synaptic plasticity: taming the beast. Nat. Neurosci..

[24] Graupner M, Wallisch P, Ostojic S (2016). Natural Firing Patterns Imply Low Sensitivity of Synaptic Plasticity to Spike Timing Compared with Firing Rate. J. Neurosci..

[25] Sjöström PJ, Turrigiano GG, Nelson SB (2003). Neocortical LTD via coincident activation of presynaptic NMDA and cannabinoid receptors. Neuron.

[26] Bender VA, Bender KJ, Brasier DJ, Feldman DE (2006). Two Coincidence Detectors for Spike Timing-Dependent Plasticity in Somatosensory Cortex. J Neurosci.

[27] Nevian T, Sakmann B (2006). Spine Ca2+ signaling in spike-timing-dependent plasticity. J. Neurosci..

[28] Banerjee A (2009). Double dissociation of spike timing-dependent potentiation and depression by subunit-preferring NMDA receptor antagonists in mouse barrel cortex. Cereb. Cortex.

[29] Fino E (2010). Distinct coincidence detectors govern the corticostriatal spike timing-dependent plasticity. J. Physiol. (Lond.).

[30] Sjöström PJ, Rancz EA, Roth A, Häusser M (2008). Dendritic excitability and synaptic plasticity. Physiol. Rev..

[31] Korte M, Schmitz D (2016). Cellular and System Biology of Memory: Timing, Molecules, and Beyond. Physiol. Rev..

[32] Pawlak V, Kerr JND (2008). Dopamine receptor activation is required for corticostriatal spike-timing-dependent plasticity. J. Neurosci..

[33] Shen W, Flajolet M, Greengard P, Surmeier DJ (2008). Dichotomous dopaminergic control of striatal synaptic plasticity. Science.

[34] Paille V (2013). GABAergic circuits control spike-timing-dependent plasticity. J. Neurosci..

[35] Valtcheva S (2017). Developmental control of spike-timing-dependent plasticity by tonic GABAergic signaling in striatum. Neuropharmacology.

[36] Fino E, Glowinski J, Venance L (2005). Bidirectional activity-dependent plasticity at corticostriatal synapses. J. Neurosci..

[37] Schulz JM, Redgrave P, Reynolds JNJ (2010). Cortico-striatal spike-timing dependent plasticity after activation of subcortical pathways. Front Synaptic Neurosci.

[38] Graupner M, Brunel N (2007). STDP in a Bistable Synapse Model Based on CaMKII and Associated Signaling Pathways. PLOS Computational Biology.

[39] Goubard V, Fino E, Venance L (2011). Contribution of astrocytic glutamate and GABA uptake to corticostriatal information processing. J. Physiol. (Lond.).

[40] Markram H, Wang Y, Tsodyks M (1998). Differential signaling via the same axon of neocortical pyramidal neurons. PNAS.

[41] Fino E, Deniau J-M, Venance L (2008). Cell-specific spike-timing-dependent plasticity in GABAergic and cholinergic interneurons in corticostriatal rat brain slices. J. Physiol. (Lond.).

[42] Froemke RC, Poo M-M, Dan Y (2005). Spike-timing-dependent synaptic plasticity depends on dendritic location. Nature.

[43] Tzounopoulos T, Rubio ME, Keen JE, Trussell LO (2007). Coactivation of Pre- and Postsynaptic Signaling Mechanisms Determines Cell-Specific Spike-Timing-Dependent Plasticity. Neuron.

[44] Xu J-Y, Zhang J, Chen C (2012). Long-lasting potentiation of hippocampal synaptic transmission by direct cortical input is mediated via endocannabinoids. J. Physiol. (Lond.).

[45] Wang, W. *et al*. A Primary Cortical Input to Hippocampus Expresses a Pathway-Specific and Endocannabinoid-Dependent Form of Long-Term Potentiation. *eNeuro* **3**, ENEURO.0160–16.2016 (2016). 10.1523/ENEURO.0160-16.2016 PMC4976302 27517090

[46] Zhu PJ, Lovinger DM (2007). Persistent synaptic activity produces long-lasting enhancement of endocannabinoid modulation and alters long-term synaptic plasticity. J. Neurophysiol..

[47] Lin Q-S (2011). Hippocampal endocannabinoids play an important role in induction of long-term potentiation and regulation of contextual fear memory formation. Brain Res. Bull..

[48] Carlson G, Wang Y, Alger BE (2002). Endocannabinoids facilitate the induction of LTP in the hippocampus. Nat. Neurosci..

[49] Maglio, L. E., Noriega-Prieto, J. A., Maraver, M. J. & Fernández de Sevilla, D. Endocannabinoid-Dependent Long-Term Potentiation of Synaptic Transmission at Rat Barrel Cortex. *Cereb. Cortex* 1–14, 10.1093/cercor/bhx053 (2017). 10.1093/cercor/bhx053 28334325

[50] Lisman, J. & Spruston, N. Questions about STDP as a General Model of Synaptic Plasticity. *Front. Synaptic Neurosci*. **2** (2010). 10.3389/fnsyn.2010.00140 PMC3059684 21423526

[51] Sjöström PJ, Turrigiano GG, Nelson SB (2004). Endocannabinoid-dependent neocortical layer-5 LTD in the absence of postsynaptic spiking. J. Neurophysiol..

[52] Fino E, Deniau J-M, Venance L (2009). Brief subthreshold events can act as Hebbian signals for long-term plasticity. PLoS ONE.

[53] Brandalise F, Gerber U (2014). Mossy fiber-evoked subthreshold responses induce timing-dependent plasticity at hippocampal CA3 recurrent synapses. Proc. Natl. Acad. Sci. USA.

[54] Dudek SM, Bear MF (1992). Homosynaptic long-term depression in area CA1 of hippocampus and effects of N-methyl-D-aspartate receptor blockade. Proc. Natl. Acad. Sci. USA.

[55] Mulkey RM, Malenka RC (1992). Mechanisms underlying induction of homosynaptic long-term depression in area CA1 of the hippocampus. Neuron.

[56] Mistry R, Dennis S, Frerking M, Mellor JR (2011). Dentate gyrus granule cell firing patterns can induce mossy fiber long-term potentiation *in vitro*. Hippocampus.

[57] Perrett SP, Dudek SM, Eagleman D, Montague PR, Friedlander MJ (2001). LTD induction in adult visual cortex: role of stimulus timing and inhibition. J. Neurosci..

[58] Massey PV (2004). Differential roles of NR2A and NR2B-containing NMDA receptors in cortical long-term potentiation and long-term depression. J. Neurosci..

[59] Remy S, Spruston N (2007). Dendritic spikes induce single-burst long-term potentiation. PNAS.

[60] Holthoff K, Kovalchuk Y, Yuste R, Konnerth A (2004). Single-shock LTD by local dendritic spikes in pyramidal neurons of mouse visual cortex. J. Physiol. (Lond.).

[61] Butts DA, Kanold PO, Shatz CJ (2007). A burst-based ‘Hebbian’ learning rule at retinogeniculate synapses links retinal waves to activity-dependent refinement. PLoS Biol..

[62] Isaac JTR, Buchanan KA, Muller RU, Mellor JR (2009). Hippocampal place cell firing patterns can induce long-term synaptic plasticity *in vitro*. J. Neurosci..

[63] Shmiel T (2005). Neurons of the cerebral cortex exhibit precise interspike timing in correspondence to behavior. PNAS.

[64] London M, Roth A, Beeren L, Häusser M, Latham PE (2010). Sensitivity to perturbations *in vivo* implies high noise and suggests rate coding in cortex. Nature.

[65] Brette, R. Philosophy of the Spike: Rate-Based vs. Spike-Based Theories of the Brain. *Front. Syst. Neurosci*. **9** (2015). 10.3389/fnsys.2015.00151 PMC4639701 26617496

[66] Zuo Y (2015). Complementary contributions of spike timing and spike rate to perceptual decisions in rat S1 and S2 cortex. Curr. Biol..

[67] Lindskog M, Kim M, Wikström MA, Blackwell KT, Kotaleski JH (2006). Transient calcium and dopamine increase PKA activity and DARPP-32 phosphorylation. PLoS Comput. Biol..

[68] Fernandez E, Schiappa R, Girault J-A, Le Novère N (2006). DARPP-32 is a robust integrator of dopamine and glutamate signals. PLoS Comput. Biol..

