# Supplementary material for: Robustness of STDP to spike timing jitter
Source: Sci Rep. 2018 May 25;8:8139. doi: 10.1038/s41598-018-26436-y (PMC5970212; doi:10.1038/s41598-018-26436-y)
Supplement: Supplementary file 1 — Supporting Information [file 41598_2018_26436_MOESM1_ESM.pdf]

Robustness of STDP to spike-timing jitter

Y. Cui, I. Prokin, A. Mendes, H. Berry, L. Venance

SUPPORTING INFORMATION

S1 Text - Description of the mathematical model

We describe here the mathematical model used to predict the outcome of STDP stimulations with spike timing jitter. We give here the information necessary to implement the model. Further detail and validation can be found in Cui et al (2016).

Stimulations. A STDP protocol consists in a series of N_{pairings} post- and presynaptic pairs of stimulations. We note t_{pre_i} the time at which the ith presynaptic stimulation is delivered and t_{post_i} the onset time of the ith postsynaptic step current stimulation, where $i = 1, \ldots, N_{\mathrm{pairings}}$. We modeled glutamate concentration in the synaptic cleft, $G(t)$, as a train of exponentially-decaying impulses triggered by presynaptic stimuli at time t_{pre_i}:

$$G(t) = G_{\max} \sum_{\mathrm{i}} \exp\left(-\frac{t-t_{\mathrm{pre}_i}}{\tau_{\mathrm{G}}}\right) \Theta\left(t - t_{\mathrm{pre}_i}\right) \qquad \text{(SI1)}$$

where $\mathrm{G_{max}}$ is the peak glutamate concentrations and τ_{G} is the glutamate clearance rate. On the postsynaptic side, action currents resulting from postsynaptic stimulations were modeled according to

$$I_{\mathrm{action}}(t) = -\mathrm{DC_{max}} \sum_{\mathrm{i}} R\left(t, t_{\mathrm{post}_i}, \mathrm{DC_{dur}}\right) - \mathrm{AP_{max}} \sum_{\mathrm{i}} \Theta\left(t - \delta - t_{\mathrm{post}_i}\right) \exp\left(\frac{-t+\delta+t_{\mathrm{post}_i}}{\tau_{\mathrm{bAP}}}\right) \text{(SI2)}$$

where the rectangle function $R(t, a, l) = \Theta(t - a) - \Theta(t - a - l)$, $\mathrm{DC_{max}}$ and $\mathrm{DC_{dur}}$ are the amplitude and duration of step-current injected by the patch pipette in the postsynaptic soma, $\mathrm{AP_{max}}$ is the amplitude of the action current producing the bAP; and τ_{bAP} is the time scale for bAP decay.

We modeled the electrical response to these stimulations in a postsynaptic element considered as a single isopotential compartment with AMPAR, NMDAR, VSCC and TRPV1 conductances

$$C_{\mathrm{m}}\frac{dV}{dt} = -g_L(V - V_{\mathrm{L}}) - I_{\mathrm{AMPAR}}(V, G(t)) - I_{\mathrm{NMDAR}}(V, G(t)) - I_{\mathrm{VSCC}}(V) - I_{\mathrm{TRPV1}}(V, AEA) - I_{\mathrm{action}}(t) \tag{SI3}$$

where V is membrane potential; g_{L} and V_{L} are leak conductance and reversal potential respectively; I_{AMPAR}, I_{NMDAR}, I_{VSCC} and I_{TRPV1} are currents through AMPAR, NMDAR, L-type VSCC (v1.3) and TRPV1, respectively, and AEA stands for anandamide. NMDAR and AMPAR were modeled with two-state kinetic models and 1.0mM Mg^{2+} (Destexhe *et al.,* 1995) whereas the model and parameters for the $Ca_v1.3$ VSCC current was taken from Wolf *et al.* (2005). The TRPV1 current, including its dependence on AEA, was modeled as:

$$I_{TRPV1}(V, AEA) = g_{TRPV1} \cdot V \cdot P_{TRPV1}^{open}(V, AEA) \tag{SI4}$$

where g_{TPRV1} is the maximal conductance of TRPV1 and the mathematical expression for the P_{TRPV1}^{open} was taken from Matta and Ahern (2007).

Biochemical signaling. Free cytosolic calcium is one of the main signaling actors in the model. To model its dynamics, we assumed it can be transferred from/to two main sources: (*i*) extracellular calcium, via the plasma membrane channels of eq. (SI3) above and (*ii*) the endoplasmic reticulum (ER), via the IP3-dependent Calcium-Induced Calcium Release (CICR) system. Hence, the concentration of free cytosolic calcium *C* was computed according to

$$T_{\mathrm{C}}(C)\frac{dC}{dt} = J_{IP_3R} - J_{\mathrm{SERCA}} + J_{\mathrm{leak}} + J_{\mathrm{NMDAR}} + J_{\mathrm{VSCC}} + J_{\mathrm{TRPV1}} - \frac{C - C_{\mathrm{b}}}{\tau_{C_{\mathrm{b}}}} \tag{SI5}$$

where the fluxes J_{IP3R}, J_{SERCA}, J_{leak} from and to the ER in the CICR system were taken from the model of De Pittà *et al.* (2009) - see also Cui et al. (2016) and J_{NMDAR}, J_{VSCC} and J_{TRPV1} are the calcium fluxes from the plasma membrane channels (eq. SI3), computed as $J_{\mathrm{x}} = \xi_x \cdot I_{\mathrm{x}}$ were the ξ_x are constants, $x \in \{\mathrm{NMDAR, VSCC, TRPV1}\}$. C_{b} is the basal cytosolic calcium level resulting from equilibration with calcium diffusion out of the cell and $\tau_{C_{\mathrm{b}}}$ is the corresponding time scale. The CICR model also includes the dynamics of the fraction of inactive IP3 (inositol triphosphate) receptor channels (IP3R), *h*

$$\frac{\mathrm{d}h}{\mathrm{d}t} = a_2 d_2 \frac{IP3 + d_1}{IP3 + d_3}(1 - h) - a_2 C h \tag{SI6}$$

Likewise, the dynamics of C_{ER}, the calcium concentration in the endoplasmic reticulum (ER) was given by

$$T_{C_{ER}}\left(C_{ER}\right)\frac{dC_{ER}}{dt}=-\rho_{ER}\left(J_{IP_3R}-J_{SERCA}+J_{leak}\right) \tag{SI7}$$

where ρ_{ER} is the ER to cytoplasm volume ratio. In eq.(SI5) and (SI7), T_x , $x = C$ or $C_{ER,}$ is a time scaling factor accounting for the presence of endogenous calcium buffers and expressed according to

$$T_x\left(x\right)=1+\frac{B_T}{K_{dB}\left(1+x/K_{dB}\right)^2} \tag{SI8}$$

Our model also accounts for the biochemical pathways leading to the production of the endocannabinoids 2-Arachidonoylglycerol (2-AG) and AEA, and their subsequent activation of cannabinoid receptors type-1, CB_1R. Principally, the model expresses the kinetics of the following enzymatic reactions: (*i*) synthesis of DAG (diacylglycerol) and IP3 (inositol triphosphate) as a result of glutamate (*G*) binding of metabotropic glutamate receptors (mGluR) and subsequent activation of PLCβ as well as Ca-dependent synthesis by PLCδ, (*ii*) transformation of DAG into 2-AG by DAG Lipase α and (*iii*) synthesis of AEA by calcium-dependent fatty acid amide hydrolase (FAAH). The dynamics of this system was modeled with the following equations:

$$\frac{dIP_3}{dt}=v_\beta\frac{G}{G+K_R+K_P C/\left(C+K_\pi\right)}+\frac{v_\delta}{1+IP_3/\kappa_d}\frac{C^2}{K_\delta^2+C^2}-v_{3K}CaMKII*\frac{IP_3^{n_3}}{K_3^{n_3}+IP_3^{n_3}}-r_{5P}IP_3 \tag{SI9}$$

$$\frac{dDAG}{dt}=v_\beta\frac{G}{G+K_R+K_P C/\left(C+K_\pi\right)}+\frac{v_\delta}{1+IP_3/\kappa_d}\frac{C^2}{K_\delta^2+C^2}-\frac{r_{DGL}\cdot DAGL\cdot\varphi_{DAGL}\cdot DAG}{DAG+K_{DAGL}}-r_{DAGK}\cdot DAG \tag{SI10}$$

$$\frac{d2AG}{dt}=\frac{r_{DGL}\cdot DAGL\cdot\varphi_{DAGL}\cdot DAG}{DAG+K_{DAGL}}-r_{MAGL}2AG \tag{SI11}$$

$$\frac{d\varphi_{DAGL}}{dt}=r_k C^{n_c}\left(1-\varphi_{DAGL}\right)-r_p\varphi_{DAGL} \tag{SI12}$$

$$\frac{dAEA}{dt}=\upsilon_{AT}C-r_{FAAH}\frac{AEA}{K_{FAAH}+AEA} \tag{SI13}$$

where *CaMKII** is the amount of activated (phosphorylated) CaMKII (see below); φ_{DAGL} is the fraction of active DAG Lipase α and DAGL its total (activated+not activated) concentration. Eq. (SI9-SI11) account for IP3 degradation by IP3 3-kinase (3K) and inositol polyphosphate 5-phosphatase (5P), while DAG and 2AG are degraded by DAG kinase (DAGK) and monoacylglycerol lipase (MAGL), respectively.

2-AG and AEA are retrograde signaling molecules that are produced in the postsynaptic neuron but diffuse to the presynaptic cell, where they activate CB_1R. We modeled CB_1R activation by 2-AG and AEA using a simple three-state kinetic model: open (x_{CB1R}), desensitized (d_{CB1R}) and inactivated (i_{CB1R}):

$$\frac{dx_{CB1R}}{dt} = \alpha_{CB1R} \cdot eCB \cdot i_{CB1R} - \left(\beta_{CB1R} + \gamma_{CB1R}\right) x_{CB1R} \tag{SI14}$$

$$\frac{dd_{CB1R}}{dt} = -\varepsilon_{CB1R} d_{CB1R} + \gamma_{CB1R} x_{CB1R} \tag{SI15}$$

where eCB = *2-AG* + 0.10 *AEA* and mass conservation implies $x_{\mathrm{CB1R}} + d_{\mathrm{CB1R}} + i_{\mathrm{CB1R}} = 1$. The open fraction x_{CB1R} was then used to compute CB_1R activation as

$$y_{\mathrm{CB1R}} = k_{\mathrm{CB1R}} x_{\mathrm{CB1R}} + C_1 \tag{SI16}$$

where C_1 is a constant that accounts for the modulation of presynaptic plasticity by other pathways and k_{CB1R} quantifies the strength of CB_1R activation on presynaptic plasticity.

In our model, CB_1R activation (y_{CB1R}) controls the presynaptic weight W_{pre} according to the following rule: W_{pre} decreases for intermediate values of y_{CB1R}, i.e. when y_{CB1R} is comprised between two tLTD thresholds ($\theta_{\mathrm{LTD}}^{\mathrm{start}} < y_{\mathrm{CB1R}} < \theta_{\mathrm{LTD}}^{\mathrm{stop}}$) whereas W_{pre} increases when y_{CB1R} is larger than a tLTP threshold ($y_{\mathrm{CB1R}} > \theta_{\mathrm{LTP}}^{\mathrm{start}}$). Following Cui et al. (2016), we implemented this rule as

$$\Omega(y_{\mathrm{CB1R}}) = \begin{cases} 1 - A_{\mathrm{LTD}} & \text{if } \Theta_{\mathrm{LTD}}^{\mathrm{start}} < y_{\mathrm{CB1R}} < \Theta_{\mathrm{LTD}}^{\mathrm{stop}} \\ 1 + A_{\mathrm{LTP}} & \text{if } \Theta_{\mathrm{LTP}}^{\mathrm{start}} < y_{\mathrm{CB1R}} \\ 1 & \text{otherwise} \end{cases} \tag{SI17}$$

and

$$\frac{dW_{\mathrm{pre}}}{dt} = \frac{\Omega(y_{\mathrm{CB1R}}) - W_{\mathrm{pre}}}{\tau_{W_{\mathrm{pre}}}(y_{\mathrm{CB1R}} + C_2)} \quad \text{(SI18)}$$

Here Ω determines the change of presynaptic plasticity, with a time scale $\tau_{W_{\mathrm{pre}}}$ set to yield rapid changes of W_{pre} for large y_{CB1R}values and very slow changes at very low y_{CB1R}:

$$\tau_{W_{\mathrm{pre}}}(\mathrm{x}) = \frac{P_1}{P_2^{P_3} + x^{P_3}} + P_4 \quad \text{(SI19)}$$

To account for experimental observation that the presynaptic weight ranges from about 50 to 300%, W_{pre} was clipped to 3.0 (hard bound).

Postsynaptic plasticity in the model was based on the activation by calcium of calmodulin and CaMKII and the regulation of this system by PKA, calcineurin and protein phosphatase 1 (PP1). The model of Cui et al (2016) for this subsection is based on the model proposed in Graupner & Brunel (2007). In this model the concentration of the calcium/calmodulin complex with four calcium ions bound (CaM) is considered at equilibrium and computed as:

$$CaM = \frac{CaMT}{1 + \sum_{i=1}^{4}(C^i \cdot \prod_{j=5-i}^{4} K_j)} \quad \text{(SI20)}$$

where *CaMT* is the total calmodulin concentration and K_i stands for the equilibrium constant of the binding of the ith calcium ion to calmodulin. Each CaMKII enzyme consists of two subunits, each of which contains 6 phosphorylation sites, thus defining 14 phosphorylation states per subunit. Denoting y_i, i=0…13 the concentrations of subunit in phosphorylation state i, the model of Graupner & Burnel (2007) expresses their dynamics as:

$$
\begin{aligned}
\frac{dy_1}{dt} &= 6k_6\gamma^2 y_0 - (4k_6\gamma^2 + k_7\gamma + k_{10})y_1 + 2k_{10}\sigma_2^4 \\
\frac{dy_2}{dt} &= (k_7\gamma + k_6\gamma^2)y_1 - (3k_6\gamma^2 + k_7\gamma + 2k_{10})y_2 + k_{10}(y_5 + \sigma_5^7) \\
\frac{dy_3}{dt} &= 2k_6\gamma^2 y_1 - 2(k_7\gamma + k_6\gamma^2 + k_{10})y_3 + k_{10}(\sigma_5^7 + 3y_8) \\
\frac{dy_4}{dt} &= k_6\gamma^2 y_1 - 2(k_7\gamma + k_6\gamma^2 + k_{10})y_4 + k_{10}(y_6 + y_7) \\
\frac{dy_5}{dt} &= k_7\gamma(\sigma_2^3 - y_5) + k_6\gamma^2(y_2 - 2y_5) + k_{10}(2y_9 + y_{10} - 3y_5) \\
\frac{dy_6}{dt} &= k_6\gamma^2(\sigma_2^3 - y_6) + k_7\gamma(2y_4 - 2y_6) + k_{10}(-3y_6 + \sigma_9^{11} + y_{11}) \\
\frac{dy_7}{dt} &= k_6\gamma^2(y_2 + 2y_4 - y_7) + k_7\gamma(y_3 - 2y_7) + k_{10}(-3y_7 + \sigma_9^{11} + y_{11}) \\
\frac{dy_8}{dt} &= k_6\gamma^2 y_3 - 3k_7\gamma y_8 + k_{10}(y_{10} - 3y_8) \\
\frac{dy_9}{dt} &= k_7\gamma(\sigma_5^7 - y_9) + k_6\gamma^2(y_5 - y_9) + k_{10}(-4y_9 + 2y_{12}) \\
\frac{dy_{10}}{dt} &= k_6\gamma^2 y_5 + k_6\gamma^2 y_6 + k_7\gamma(y_7 + 3y_8 - 2y_{10}) + k_{10}(2y_{12} - 4y_{10}) \\
\frac{dy_{11}}{dt} &= k_7\gamma(y_6 - 2y_{11}) + k_6\gamma^2 y_7 + k_{10}(y_{12} - 4y_{11}) \\
\frac{dy_{12}}{dt} &= k_6\gamma^2 y_9 + k_7\gamma(2\sigma_9^{11} - y_9 - y_{12}) + k_{10}(6y_{13} - 5y_{12}) \\
\frac{dy_{13}}{dt} &= k_7\gamma y_{12} - 6k_{10}y_{13}
\end{aligned}
\qquad \text{(SI21)}
$$

where $\sigma_i^j = \sum_{k=i}^{j} y_k$ and mass conservation implies $y_0 = 2CaMKII_0 - \sigma_1^{13}$, where $CaMKII_0$ is the total CaMKII concentration. In eq. (SI21) above the probabilities that CaM binds to phosphorylated (γ) and dephosphorylated (γ^*) subunit of CaMKII are computed as $\gamma = CaM/(CaM + K_5)$ and $\gamma^* = CaM/(CaM + K_9)$. Moreover, the rate of subunit dephosphorylation (k_{10}) is given by $k_{10} = k_{12}PP1/(K_M + CaMKII^*)$ where K_M is a constant, $PP1$ is the concentration of PP1. $CaMKII^*$ is the total concentration of phosphorylated subunits of CaMKII computed accross all possible states of phosphorylation $CaMKII^* = \sum_{i=0}^{13} m_i y_i$ where m_i is the number of the phosphorylated subunits of CaMKII in state i and y_i.

Finally, the model of Graupner & Brunel (2007) assumes that, in addition to calcineurin (CaN), PKA activity depends on CaM according to a Hill-function activation:

$$
v_x(CaM) = k_x^0 + \frac{k_x}{1+(K_x/CaM)^{nx}} \qquad \text{(SI22)}
$$

where $x = CaN$ or PKA, The interaction between PP1 and I1 is then described as:

$$
\begin{aligned}
\frac{dI1}{dt} &= \frac{dPP1}{dt} - v_{CaN}I1 + v_{PKA}I1_0 \\
\frac{dPP1}{dt} &= -k_{13}I1 \cdot PP1 + k_{-13}(PP1_0 - PP1)
\end{aligned}
\qquad \text{(SI23)}
$$

where $PP1_0$ and $I1_0$ are total PP1 and I1 concentrations, respectively.

In agreement with Cui et al. (2016) and Graupner & Brunel (2007), we assumed that postsynaptic plasticity is directly proportional to the calcium-dependent activation of CaMKII and set:

$$W_{\text{post}} = 1 + 3.5\frac{CaMKII^*}{CaMKII^*_{\text{max}}} \tag{SI24}$$

where $CaMKII^*_{\text{max}}$ is the maximal concentration of activate (phosphorylated).

Finally, the total synaptic weight was taken as the product of the presynaptic and the postsynaptic contributions above:

$$W_{\text{total}} = W_{\text{pre}}W_{\text{post}} \tag{SI25}$$

Model implementation and numerics. Our mathematical model comprises 36 ordinary differential equations and close to 150 parameters, among which more than one half is constrained by experimental data. Initial conditions were set to the steady-state of each variable in the absence of stimulation. Numerical solution was obtained with the LSODA solver from the ODEPACK fortran77 library with absolute and relative tolerances both equal to 10^{-7}. Initial conditions were set to the steady-state of each variable in the absence of stimulation. Numerical integration proceeded until the synaptic weights reach stable values (typically observed around t ≈ 5min after the stimulation protocol). We used the final values of the pre- and postsynaptic weights to compute the total synaptic weight change due to the stimulations. Importantly, with the exception of the stimulation protocols, *we have used the exact same equations and parameter values as in* Cui et al. (2016). The list of parameters and their estimated values is given in S2 Table. The current study employs stochastic simulations since the stimulation protocol is stochastic (while the rest of the model is deterministic). Therefore, the model was calibrated using experimental data from deterministic stimulation

protocols (Cui et al., 2016) and we tested here whether it can make successful predictions when we applied stochastic stimulation protocols, for which the model was not calibrated. The results are thus averages over N_{trials} realizations. N_{trials} was varied from 30 to 500 depending on the smoothness of the averaged curves and values of SEM, but N_{trials}=50 in most of the simulations. The computer code of the model is available online from https://github.com/iprokin/Cx-Str-STDP and https://senselab.med.yale.edu/modeldb/ShowModel.cshtml?model=187605.

S2 Table – Parameter values

A. Intracellular dynamics

Description	Name	Values	Units	Reference
I_{NMDAR} to Ca flow conversion	ξ_{NMDAR}	98	µM/pCol	set to match Ca2+ amplitudes in [1]
I_{VSCC} to Ca flow conversion	ξ_{VSCC}	140	µM/pCol	set to match Ca2+ amplitudes in [2]
I_{TRPV1} to Ca flow conversion	ξ_{TRPV1}	290	µM/pCol	set to match Ca2+ amplitudes in [1]
Total endogenous Ca buffer	B_T	4.5	µM	Estimated from our experimental data
Basal cytoplasmic [Ca]	C_b	0.1	µM	[1,3]
Time scale for Ca exit	τ_{Cb}	0.007	s	Idem
Endogenous Ca buffer affinity	K_{dB}	0.5	µM	[3,4]
ER-to-cytosol volume ratio	ρ_{ER}	0.3	-	Adapted from [4]
IP3R binding rate (inactivation)	a_2	0.5	µM/s	Idem
Maximal SERCA pump rate	v_{ER}	8	µM/s	Idem
IP3R affinity for IP3	d_3	0.9434	µM	[5]
Maximal IP3R rate	r_C	4	1/s	Adapted from [5]
Basal [Ca] in the ER	$Ca_{ER,b}$	65	µM	[6]
Ca leak from the ER	r_l	0.1	1/s	[5]
IP3R affinity for Ca	d_5	0.12	µM	Adapted from [5]
IP3R dissociation constant	d_2	3.049	µM	Idem
SERCA pump affinity for Ca	K_{ER}	0.05	µM	[5]
IP3R affinity for IP3	d_1	0.13	µM	Idem
PLCδ product inhibition	κ_d	1.5	µM	Idem
PLCδ Ca-activation	K_δ	0.1	µM	Idem
5P-IP maximal rate	r_{5P}	0.2	1/s	Adapted from [5]
PI3K maximal rate	v_{3K}	0.001	µM/s	Idem
PI3K Ca-activation constant	K_D	1.5	µM	Idem
PLCδ maximal rate	v_δ	0.02	µM/s	[5]
PI3K affinity for IP3	K_3	1	µM	Idem
Glutamate affinity to mGluR	K_R	1.3	µM	Idem
regulation by PLCβ termination	K_P	10	µM	Idem
PLCβ maximal rate	v_β	0.8	µM/s	Adapted from [5]
PKC Ca-activation constant	K_π	0.6	µM	[5]
Total CaMKIIα concentration	$CaMKII_0$	16.6	µM	[7]
Total Calmodulin concentration	$CaMT$	0.07085	µM	Adapted from [7]
PKA Hill number	n_{PKA}	3	-	Idem

Referenced articles: [1] Sabatini, B.L., Oertner, T.O. and Svoboda, K. (2002) *Neuron* 33(3):439-452 [2] Carter, A.G. and Sabatini, B.L. (2004) *Neuron* 44(3):483-493 [3] Jackson, M.B. and Redman, S.J. (2003) *J Neurosci* 23:1612–1621 [4] Nägerl, U.V. *et al.* (2000) *Biophys J* 79: 3009–3018 [5] De Pittà M. *et al.* (2009) *J Biol Phys* 35:383-411 [6] Solovyova, N. *et al.* (2002) *EMBO J* 21:622-630 [7] Graupner, M. and Brunel, N. (2007) *PLoS Comput Biol* 3:e221.

B. Electrophysiology

Description	Name	Values	Units	Reference
TRPV1R max. conductance	g_{TRPV1}	0.0003	nS	Estimated from our experimental data
Permeability of L-type VSCC	p_{VSCC}	0.00000102	μM/s	Value of [1], scaled to ~5,000 spines, radius 1 μm
AMPAR maximal conductance	g_{AMPAR}	5.1	nS	Estimated from our experimental data
AMPAR closing rate constant	β_{AMPAR}	190	1/s	Adapted from [2]
AMPAR opening rate constant	α_{AMPAR}	1.02	1/(μM.s)	Idem
NMDAR maximal conductance	g_{NMDAR}	1.53	nS	Estimated from our experimental data
Magnesium concentration	Mg	1	mM	Directly measured in our experiments
NMDAR opening rate constant	α_{NMDAR}	0.072	1/(μM.s)	Set to emulate the experimental kinetics of NMDAR-transported Ca in [3]
NMDAR closing rate constant	β_{NMDAR}	100	1/s	Idem
Resting membrane potential	V_L	-70	mV	Directly measured in our experiments
Leak conductance	g_L	10	nS	Idem
Membrane capacitance	C_m	0.1	nF	Idem
Extracellular Ca2+ concentration	Ca_{out}	5000	μM	[1]
Duration of depolarization step	DC_{dur}	0.03	s	From the experimental stimulation protocol
Amplitude of depolarization step	$DC_{\max}$	495	pA	Idem
Amplitude of action current	$AP_{\max}$	7020	pA	[4]
Time constant of action current	τ_{bAP}	0.001	s	Idem
Glutamate peak concentration in the cleft	$G_{\max}$	2000	μM	Estimated from our experimental data
Glutamate decay time constant in the cleft	τ_G	0.005	s	Estimated from our experimental data
Delay to bAP outset	δ	0.015	s	From the experimental stimulation protocol
Stimulation frequency	F_{pairings}	1	Hz	From the experimental stimulation protocol

Referenced articles: [1] Wolf, J.A. *et al.* (2005) *J Neurosci* 25(40):9080-9095 [2] Destexhe, A., Mainen, Z., and Sejnowski, T. (1994) *J Comput Neurosci* 1:195-230 [3] Sabatini, B.L., Oertner, T.O. and Svoboda, K. (2002) *Neuron* 33(3):439-452 [4] Fino, E., *et al.* (2010) *J Physiol* 588:3045-3062.

C. Endocannabinoid dynamics

Description	Name	Values	Units	Reference
Scaling factor for endocannabinoid contribution to plasticity	k_{CB1R}	3000	1/µM	Estimated from our experimental data
presynaptic plasticity time scale	P_1	1e-9	s	Set to yield rapid / slow changes of Wpre for high / low 2-AG values, respectively
presynaptic plasticity time scale	P_2	1e-5	-	Idem
presynaptic plasticity time scale	P_3	7	-	Idem
presynaptic plasticity time scale	P_4	2	s	Idem
The lower limit of LTD induction	θ_{LTD}^{start}	0.027	-	Estimated from our experimental data
The upper limit of LTD induction	θ_{LTD}^{stop}	0.047	-	Idem
The constant determining the rate of LTD induction	A_{LTD}	0.65	-	Idem
The lower limit of LTP induction	θ_{LTP}^{start}	0.087	-	Idem
The constant determining the rate of LTP induction	A_{LTP}	10.8	-	Idem
CB1R opening rate constant	α_{CB1R}	0.2402	1/(µM.s)	Idem
CB1R closing rate constant	β_{CB1R}	11.072	1/s	Idem
CB1R desensitization rate constant	γ_{CB1R}	416.38	1/s	Idem
CB1R closing rate constant	ε_{CB1R}	0.047796	1/s	Idem
DAGL α affinity for DAG	K_{DAGL}	30	µM	[1]
Maximal rate of MAG lipase	r_{MAGL}	0.5	µM/s	Set for rapid turnover dynamics.
Maximal DAGL α rate	r_{DGL}	20000	µM/s	Idem
DAG kinase rate	r_{DAGK}	2	s^{-1}	Idem
Hill number for DAGL activation by Ca	n_c	6	-	Estimated from our experimental data
DAGL dephosphorylation rate	r_P	380	s^{-1}	Idem
DAGL phosphorylation rate	r_K	50	$\mu M^{-1}.s^{-1}$	Idem
Total DAGL concentration	$DAGL$	1	µM	Idem
N-acetyltransferase activity	v_{AT}	0.2	1/s	Idem
FAAH Michaelis-Menten constant	K_{FAAH}	1	µM	[1]
FAAH enzyme activity	r_{FAAH}	4	µM/s	Estimated from our experimental data

Referenced articles: [1] Okamoto, Y. et *al.* (2004) *J Biol Chem* 279:5298-5305.

S3 Text – Mechanisms of STDP expression in the model

We give here an overview of the mechanisms that give rise to the expression of eCB-tLTP, eCB-tLTD and NMDAR-tLTP in our mathematical model. All the examples given here concern regular (deterministic) STDP protocols with constant IPI and spike timings.

Our model combines the two signaling pathways involved in cortico-striatal STDP: a first signaling pathway leading from NMDAR to calmodulin and CaMKII with a second that couples mGluR and cytosolic calcium to eCB production and the resulting activation of CB_1R (see Fig. 1f1). In the model, we assume that the total synaptic weight (W_{Total}) is the product of presynaptic (W_{Pre}) and postsynaptic (W_{Post}) weights (see Supporting Information S1).

On the postsynaptic side, W_{Post} is proportional to the amount of CaMKII activated by the NMDAR pathway. CaMKII in our model forms a bistable system that settles at long times either on a "DOWN" state where CaMKII is mostly inactive (no plasticity) or on an "UP" state characterized by high levels of activated CaMKII (Fig. 2b1) (NMDAR-tLTP). In the example of the figure, the time needed for CaMKII dephosphorylation is larger than the interval between two successive pairings, so activated CaMKII accumulates with the number of pairings during the protocol. For a transition from the UP to the DOWN state to occur, the amount of activated CaMKII needs to overcome the separatrix between the two states. The separatrix is reached in the example of *Supplementary Figure S2* (in Supporting Information S4 below) for circa 50-60 post-pre pairings (with Δt_{STDP} =-15 ms) (Fig. S2a1). Therefore, in the conditions of Figure S2 (short post-pre pairings), W_{Post} converges to the UP state (potentiation) only when the STDP protocol comprises > 50 post-pre pairings. With pre-post pairings, the calcium released after each pairing activates less calmodulin than with post-pre protocols. As a result, activated CaMKII never reaches the threshold for the UP state (Fig. S2a2). To summarize, the model successfully reproduces the experimental observations that (*i*) NMDAR-dependent tLTP emerges after 50-75 short post-pre pairings and (*ii*) no NMDAR-dependent LTP is obtained with pre-post pairings (Cui et al., 2016).

When a stimulation protocol composed of 100 pairings is applied, the evolution of the amplitude of the triggered calcium peaks is biphasic (Fig. S2c): it increases up to 10-20 pairings and decreases afterwards to settle to constant amplitude after circa 50 pairings. For the first 10-20 stimulations, the iterated activation of mGluRs leads to the accumulation of IP3, which contributes an extra influx of calcium from the endoplasmic reticulum through calcium-induced calcium release. This extra-boost of cytoplasmic calcium however vanishes when the number of pairings increases further because the concentration of calcium in the ER

decreases. This decay ER calcium eventually compensates the effect of IP3 accumulation on the calcium-induced calcium release, thus stabilizing the amplitude of the calcium peaks for N_{pairings}>50. Moreover, since the width of the postsynaptic calcium peak in the model is larger with post-pre than pre-post pairings (Fig S2b), the fraction of calcium-activated DAGLα is much larger for post-pre pairings. As a result, the amplitude of eCB peaks and, ultimately, the amplitude of the fraction of activated CB_1R (y_{CB1R}), show a more pronounced biphasic profile (Fig S2d). The biphasic trend is further amplified at the level of CB_1R activation because of CB_1R receptor desensitization that amplifies the decay above 20 pairings (Fig S2d). The amplitude of the y_{CB1R} peaks increases sharply for the first 10-20 pairings, and progressively decays afterwards to converge to constant amplitude. As show in the figure, y_{CB1R} reaches large values only for short post-pre pairings (Δt_{STDP} around -15 ms) while even short pre-post pairings (0< Δt_{STDP} <10 ms) do not give rise to such large amplitude peaks.

The dynamics of the level of y_{CB1R} is responsible for the emergence of eCB-dependent plasticity in the model. Indeed, in the model, W_{Pre} depends on y_{CB1R} with the piecewise constant function shown in Figure S2e and its 3 thresholds $\Theta_{\mathrm{LTD}}^{\mathrm{start}}$, $\Theta_{\mathrm{LTD}}^{\mathrm{stop}}$ and $\Theta_{\mathrm{LTP}}^{\mathrm{start}}$: if y_{CB1R} reaches moderate amounts, i.e. lies within [$\Theta_{\mathrm{LTD}}^{\mathrm{start}}$, $\Theta_{\mathrm{LTD}}^{\mathrm{stop}}$], W_{Pre} decays (thus emulating eCB-tLTD); whereas W_{Pre} increases (eCB-tLTP) if $y_{\mathrm{CB1R}} > \Theta_{\mathrm{LTP}}^{\mathrm{start}}$ (see the dashed lines in Fig. S2d and summary in Fig. S2e). With short pre-post pairings (10 < Δt_{STDP} <40 ms), y_{CB1R} enters the LTD range during most of protocol so each pairing contributes a increment of W_{Pre} reduction. Since W_{Post} is not changed with positive, pre-post pairings (Fig. S2a2), pre-post pairings globally lead to a progressive reduction of W_{Total}, i.e. the expression of eCB-tLTP. The situation is different for post-pre pairings. The amplitude of y_{CB1R} peaks overcomes $\Theta_{\mathrm{LTP}}^{\mathrm{start}}$ for ≈ 5 to 30 post-pre-pairings with short Δt_{STDP} (around -15 ms), yielding a strong increase of W_{Pre}. Given that more that 50 post-pre pairings are needed to alter W_{Post} (Fig. S2a1), the expansion of W_{Pre} leads to eCB-tLTP. However, when N_{pairings} > 30 post-pre pairings, y_{CB1R} amplitude gets back below $\Theta_{\mathrm{LTP}}^{\mathrm{start}}$ so that the initial increase of W_{Pre} does not continue beyond 30 post-pre pairings. This property of the model dynamics emulated the experimental observation that eCB-tLTP disappears when the number of pairings becomes larger than 25-30.

Therefore, the mechanisms that lead to the expression of eCB-dependent plasticity with regular deterministic stimulation protocols in our model can be summarized as follow. eCB-tLTD requires moderate levels of CB_1R activation that are reached with pre-post but not pre-post pairings. The expression of eCB-tLTP however demands larger amounts of CB_1R

activation, which are reached only during the first 5-30 post-pre pairings, where every component of the system contributes maximally to CB_1R activation. When the number of post-pre pairings becomes larger than 30, calcium efflux from the internal calcium stores decreases and CB_1R desensitization increases: CB_1R activation becomes insufficient to support such high values of CB_1R activation, so eCB-tLTP vanishes.

S4 Figure – Supplementary Figures

Figure S1

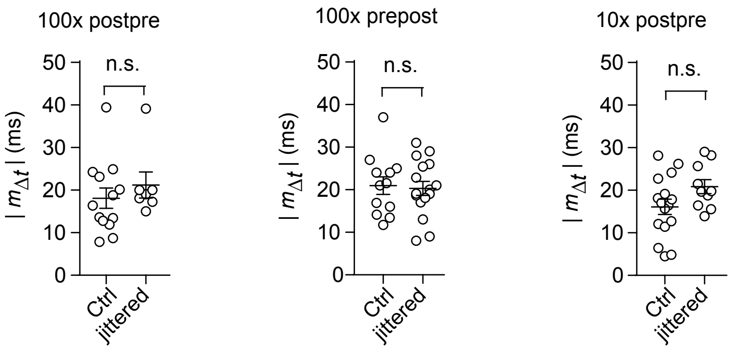

Supplementary Figure 1: $m_{\Delta t}$ values were not significantly different among STDP paradigms. $m_{\Delta t}$ were not significantly different in control and jittered conditions for 100 post-pre, 100 pre-post and 10 post-pre pairings (empty circles: individual neurons; black circle: average). Error bars represent the SEM. ns: not significant.

Figure S2

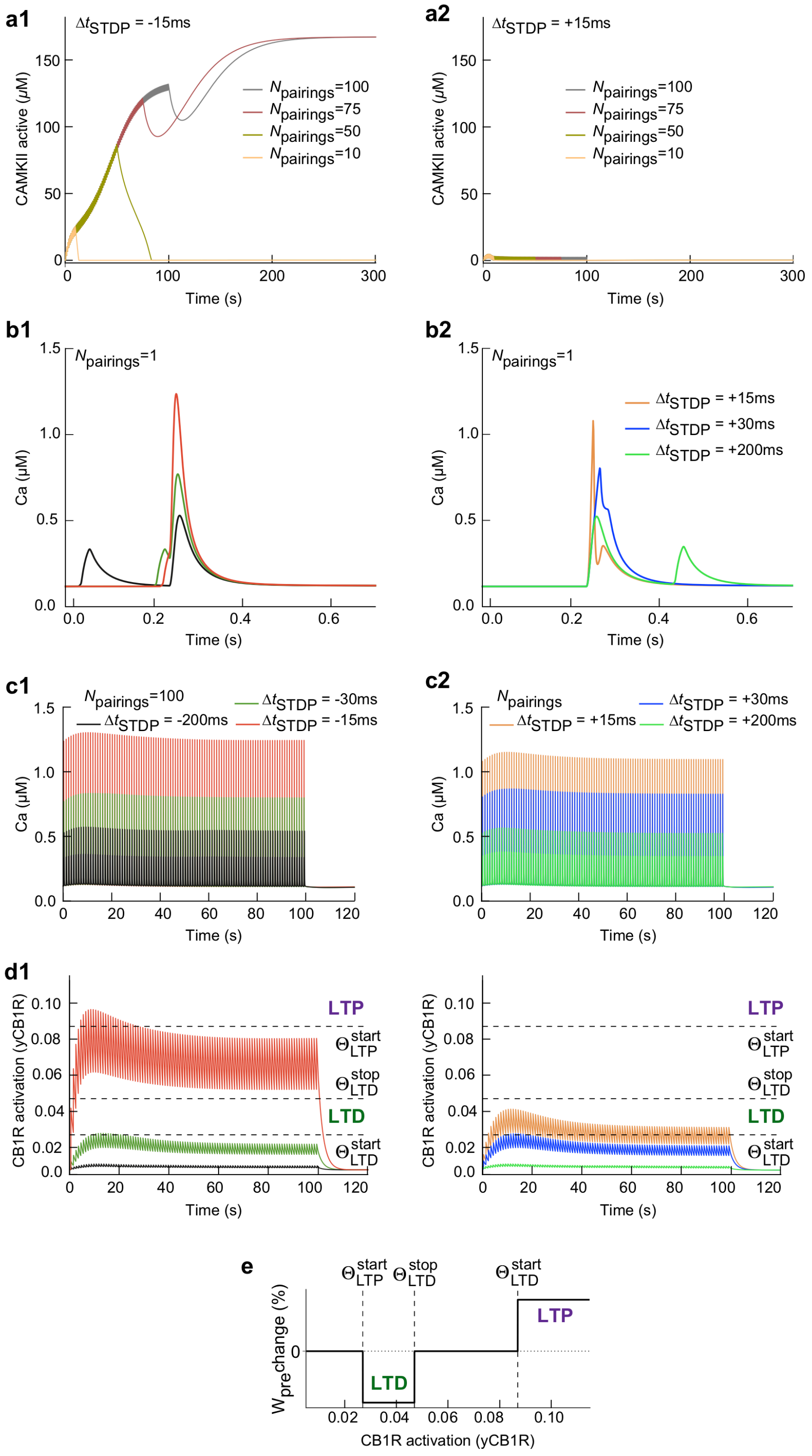

Supplementary Figure 2: **Mechanisms of STDP expression in the model with deterministic stimulation protocols**.

(**a**) Changes in the amount of active CaMKII starting from the down (non-activated) state. The number of pairings, N_{pairings}, is indicated for spike-timing Δt_{STDP} = -15 (**a1**) or + 15 (**a2**) ms. (**b**) Intracellular calcium changes for the first pairing in post-pre (**b1**) or pre-post (**b2**) pairing protocols. (**c**) Predicted dynamics of the cytoplasmic calcium for 100 post-pre (**c1**) or 100 pre-post pairings (**c2**) and corresponding activation of CB1R, y_{CB1R} (**d**). The spike-timing Δt_{STDP} for each curve is indicated in the legend. (**e**) The presynaptic weight W_{Pre} changes as a function of y_{CB1R}: it decreases (LTD) when y_{CB1R} reaches intermediate values and it increases (LTP) if y_{CB1R} overcomes the LTP threshold. The corresponding thresholds are reported in D as dashed lines. For all the simulations shown, the stimulation protocols were deterministic (regular) with a frequency of 1Hz.

SUPPLEMENTARY REFERENCES

Cui Y, Prokin I, Xu H, Delord B, Genet S, Venance L, Berry H. (2016) Endocannabinoid dynamics gate spike-timing dependent depression and potentiation. eLife **5**:1–32.

De Pittà M, Goldberg M, Volman V, Berry H, Ben-Jacob E. (2009) Glutamate regulation of calcium and IP3 oscillating and pulsating dynamics in astrocytes. J Biol Phys **35**:383–411.

Destexhe A, Mainen ZF, Sejnowski TJ (1995) Fast kinetic models for simulating AMPA, NMDA, GABA a and GABA B receptors. The Neurobiology of Computation:9–14.

Graupner M, Brunel N (2007) STDP in a bistable synapse model based on CaMKII and associated signaling pathways. PLoS Comput Biol **3**:e221.

Matta JA, Ahern GP (2007) Voltage is a partial activator of rat thermosensitive TRP channels. J Physiol **585**:469–482.

Wolf JA, Moyer JT, Lazarewicz MT, Contreras D, Benoit-Marand M, O'Donnell P, Finkel LH (2005) NMDA/AMPA ratio impacts state transitions and entrainment to oscillations in a computational model of the nucleus accumbens medium spiny projection neuron. J Neurosci **25**:9080–9095.
